# Associations of food processing and dietary risk factors with colorectal cancer incidence in the NIH-AARP Diet and Health Study: A cohort study

**DOI:** 10.1371/journal.pmed.1005251

**Published:** 2026-10-15

**Authors:** Leila Abar, Caitlin P. O’Connell, Hyokyoung G. Hong, Kirsten A. Herrick, Lisa Kahle, Jennifer L. Lerman, Linda M. Liao, Xuehong Zhang, Xinyuan Zhang, Longgang Zhao, Sémi Zouiouich, Rashmi Sinha, Neha Khandpur, Eurídice Martínez Steele, Erikka Loftfield

**Affiliations:** 1 Metabolic Epidemiology Branch, Division of Cancer Epidemiology and Genetics, National Cancer Institute, National Institutes of Health, Rockville, Maryland, United States of America; 2 Biostatistics Branch, Division of Cancer Epidemiology and Genetics, National Cancer Institute, National Institutes of Health, Rockville, Maryland, United States of America; 3 Risk Factor Assessment Branch, Division of Cancer Control and Population Sciences, National Cancer Institute, National Institutes of Health, Rockville, Maryland, United States of America; 4 Information Management Services (IMS), Inc., Calverton, Maryland, United States of America; 5 Yale School of Nursing, Yale University, New Haven, Connecticut, United States of America; 6 Channing Division of Network Medicine, Brigham and Women’s Hospital and Harvard Medical School, Boston, Massachusetts, United States of America; 7 Nutrition and Metabolism Branch, International Agency for Research on Cancer, World Health Organization, Lyon, France; 8 Division of Human Nutrition and Health, Wageningen University, Wageningen, Netherlands; 9 Centre for Epidemiological Studies in Health and Nutrition (NUPENS), University of São Paulo, São Paulo, Brazil; 10 Department of Nutrition, School of Public Health, University of São Paulo, São Paulo, Brazil; 11 Population Health Program, QIMR Berghofer Medical Research Institute, Brisbane, Australia; University of Cambridge, UNITED KINGDOM OF GREAT BRITAIN AND NORTHERN IRELAND

## Abstract

**Background:**

Ultra-processed foods (UPF) account for >50% of energy intake by US adults. UPF intake can include dietary components linked to lower colorectal cancer (CRC) risk, such as whole grain and dairy products, and others linked to higher CRC risk, such as processed meats, potentially explaining the heterogeneity of evidence for an association between UPF and CRC risk. Our goal was to estimate the associations of UPF and established CRC-related dietary factors, by UPF and non-UPF source, with CRC incidence in a large cohort study.

**Methods and findings:**

US adults, aged 50–71, who enrolled in the NIH-AARP Diet and Health Study in 1995–96, self-reported dietary intake using a food frequency questionnaire (FFQ). Median UPF intake was 293 g/1000 kcal/day or 43% of daily energy intake. We assigned disaggregated FFQ items to Nova classification and categorized UPF intake (g/1000 kcal/day) into sex-specific quintiles. Participants who were cancer-free at baseline (*N* = 461,682) were followed for cancer incidence through 2018, and 10,075 colorectal adenocarcinoma cases were diagnosed. We used multivariable-adjusted Cox proportional hazards regression models, adjusted for demographic, lifestyle, and health-related factors, including age, sex, race/ethnicity, education, physical activity, smoking, and alcohol drinking, to estimate associations for UPF and related dietary components with CRC incidence. UPF intake was not associated with CRC incidence (hazard ratio _5th versus 1st quintile_ 0.97; 95% confidence interval [0.91–1.03]; *p*_*trend*_ = 0.55). Whole grain, fiber, dairy, and calcium intakes from UPF sources were inversely but meat intake from UPF sources was positively associated with CRC incidence. The main limitations of the study relate to its observational design and include potential residual confounding and exposure misclassification owing to systematic or random error in self-reported dietary data.

**Conclusions:**

Total UPF intake was not associated with incident CRC in this cohort of older, US adults. This may be explained, in part, by opposing associations of some UPF with CRC risk. Our findings support current dietary guidance to consume whole grains, fiber, dairy, and calcium and avoid processed meat for CRC prevention.

## Author summary

**Why was this study done?:**

- Prospective studies of ultra-processed food (UPF) intake and colorectal cancer (CRC) incidence have yielded mixed results.
- UPF represent a heterogeneous array of foods—including some, such as whole grain and dairy products, that have been associated with lower CRC risk, and others, such as processed meat products, that have been associated with higher CRC risk.

**What did the researchers do and find?:**

- We analyzed data from 461,682 participants who were cancer-free at baseline in the NIH-AARP Diet and Health Study, among whom 10,075 colorectal adenocarcinoma cases were diagnosed during follow-up.
- We estimated associations between CRC incidence and total UPF intake, UPF subgroups, and key dietary factors (dairy, whole grain, and meat) from UPF and non-UPF sources.
- Total UPF intake was not associated with CRC incidence.
- Whole grain, fiber, dairy, and calcium intakes from UPF sources were associated with lower CRC incidence; meat intake from UPF sources was associated with higher CRC incidence.

**What do these findings mean?:**

- Our findings support current evidence-based dietary guidance to consume whole grains, fiber, dairy, and calcium and to avoid processed meat for CRC prevention.
- Our findings do not address whether dietary components from UPF and non-UPF sources are biologically equivalent and should be interpreted with caution given limitations of the observational design.

## Abstract 3

In this study, Leila Abar and colleagues examine ultra-processed food intake and colorectal cancer incidence in a large prospective cohort, finding no overall association and suggesting that the nutritional composition of these foods may be more relevant than their level of processing alone.

## Introduction

Colorectal cancer (CRC) is the third most commonly diagnosed cancer in the United States (US) [1], and dietary intake [2–4] has been linked to CRC development. The 2018 World Cancer Research Fund Report concluded that higher processed meat, red meat, and alcohol intake are associated with higher CRC risk, while higher whole grain, fiber, dairy, and calcium intake are associated with lower CRC risk [5]. Consumption of ultra-processed foods (UPF; Nova group 4) has been associated with higher CRC incidence in some but not all prospective cohort studies [6–10], and it remains unclear how current evidence-based dietary guidance for CRC prevention intersects with research on food processing.

Evidence- based dietary guidelines also recommend limiting intake of added sugar, sodium, and saturated fat [11–13] and prioritizing whole foods to improve health [14,15]. While most UPF are energy dense and nutrient poor [11–13], some are not (e.g., whole wheat bread). A 2023 study demonstrated that it is possible, though perhaps not practical [16], for dietary patterns high in UPF to receive high diet quality scores [17]. Additionally, prior research has shown both positive and inverse associations with certain UPF subgroups and health outcomes, including incident type 2 diabetes [18] and CRC [6]. Therefore, we sought to explore whether diet quality or food and nutrient intake impact associations of UPF intake with CRC incidence.

Our primary aim was to estimate the association between UPF intake and CRC incidence. Our secondary aims were to evaluate the joint effect of UPF intake and diet quality, measured using the Healthy Eating Index-2020 (HEI-2020) [19], with CRC incidence and to estimate associations for UPF subgroups as well as for food group (e.g., whole grain) and nutrient (e.g., fiber) intake by UPF and non-UPF source. Our hypothesis was that higher UPF intake is associated with higher CRC incidence but that it varies by diet quality, by UPF subgroup, and, for nutrient and food group analyses, by UPF versus non-UPF source.

## Methods

### Study population

The design of the National Institutes of Health (NIH)-AARP Diet and Health Study has been detailed elsewhere [20]. In brief, NIH-AARP participants, aged 50–71 years, were recruited in 1995−96 from among 3.5 million AARP members residing in one of six states (California, Florida, Louisiana, New Jersey, North Carolina, and Pennsylvania) or two metropolitan areas (Atlanta, GA and Detroit, MI). Participants self-reported information on demographic, lifestyle, and other health-related characteristics via a baseline questionnaire; 566,398 participants completed the questionnaire, which was considered to imply informed consent. The NIH-AARP Study was approved by the Special Studies NIH Institutional Review Board of the National Cancer Institute; the current study is a secondary analysis of de-identified data and was deemed exempt from further review.

We excluded participants who had a proxy respondent (*n* = 15,760); registry confirmed or self-reported cancer, except for nonmelanoma skin cancer (*n* = 51,062), end-stage renal disease (*n* = 769), or poor self-rated health (*n* = 8,365) at baseline; had a death record for cancer without a registry-confirmed cancer (*n* = 14,113); were caloric outliers, defined as >2 interquartile range (IQR) above the 75th percentile or below the 25th percentile of sex-specific Box-Cox-transformed intake (*n* = 3,664) [21,22]; or had <1 year of follow-up (*n* = 10,983). Our final analytic sample included 461,682 participants.

### Exposure assessment

Dietary intake was assessed using a food frequency questionnaire (FFQ) that included 124 food and beverage items, with portion sizes and 21 questions on low-fat, high-fiber foods and food preparation methods. Methods used to convert FFQ responses into USDA food codes [23] and classify them according to Nova [24] have been described in detail elsewhere. Briefly, FFQ line items were comprised of 3,513 individual food codes using the USDA’s 1994–1996 Continuing Survey of Food Intake of Individuals (CSFII). CSFII food codes were matched 1:1 to 8-digit USDA Food and Nutrient Database for Dietary Studies (FNDDS) food codes, representing the most commonly consumed foods and beverages at the population level, which were unfolded into component 8-digit food codes and 3,553 unique standard reference (SR) codes [24]. Each SR code was classified according to Nova through database linkage; gram weights and energy values were each summed to the parent food code level [23,24].

To estimate intake of food groups and nutrients according to Nova, we used the Food Patterns Ingredient Database (FPID), 2005−2006 through 2017−18, matching each SR code to its first occurrence in FPID; 192 SR codes that could not be directly matched in FPID databases were reviewed and assigned a proxy SR code based on descriptions that included information about whether a product was ready-to-eat/drink (e.g., “14323–orange drink, canned” was assigned as “14435–orange breakfast drink, ready-to-drink”). It is important to note that the 37 USDA Food Patterns (FP) components that can be estimated using FPID, operationalize unprocessed red meat and processed (red and white) meat as mutually exclusive components. For this reason, our analysis focuses on total meat by Nova classification.

The estimation and validation of gram weight and energy intake according to Nova in the NIH-AARP calibration sub-study has been described previously [24]. In brief, validity coefficients, which if close to 0 indicate severe misclassification and if close to 1 indicate little misclassification, for absolute energy and gram weight intake from UPF were 0.43 and 0.59 for men and 0.33 and 0.59 for women, respectively, before adjustment for total energy intake. After energy adjustment, validity coefficients for gram weight intake from UPF increased to 0.66 for both men and women [23,24]. Because energy-adjusted gram weight UPF intake outperformed UPF intake based on grams or energy alone, our primary exposure variable was sex-specific quintiles of energy-adjusted UPF intake (g/1000 kcal/day). We used the nutrient-density method to adjust for total energy [25]. We also created sex-specific quintiles using percentage of total energy (% kcal/day) and grams (% g/day) from UPF. We decided *a priori* to adjust for alcoholic beverage intake, so grams or calories from alcoholic beverages were not included in Nova variables [26].

HEI-2020, a diet quality index that measures alignment with the Dietary Guidelines for Americans 2020−2025 uses the same components and scoring standards as the HEI-2020; estimation in the NIH-AARP Study has been described in detail elsewhere [19].

### Cohort follow-up and case ascertainment

Incident cancer cases were identified through cancer registry linkage in study states and three states popular for relocation (Arizona, Nevada, and Texas). Vital status was determined through National Death Index linkage. Participant relocation was assessed annually by linkage to the National Change of Address database maintained by the US Postal Service, through receipt of US Postal Service processing of undeliverable mail, from other address change update services, and directly from participants who report address changes in response to study mailings. Prior research to determine the completeness of cancer case ascertainment in the NIH-AARP cohort demonstrated that state cancer registries captured more than 90% of incident cases when compared with Surveillance Epidemiology and End Results estimates [27]. Follow-up time was defined from baseline to date of cancer diagnosis, death, relocation outside catchment area, or end of study follow-up (December 31, 2018), whichever occurred first. Exit age was calculated based on entry age and duration of follow-up.

CRC cases were identified using the International Classification of Diseases for Oncology, Third Edition (ICD-O-3). We restricted our definition to primary adenocarcinoma of the proximal colon (C180, C182, C183, and C184), distal colon (C185, C186, C187), and rectum (C199, C209) using the following histology codes: 8140, 8141, 8143, 8145, 8210, 8211, 8221, 8260, 8261, 8262, 8263, 8470, 8480, 8481, and 8490. Cases with overlapping colon lesions (C188), unspecified colon cancer (C189), and large intestine not otherwise specified (C260) were censored at their date of diagnosis in subsite analyses.

### Statistical analysis

We summarized baseline characteristics by study inclusion and quintile of UPF intake. We used Cox proportional hazards regression models, with age as the underlying time metric, to estimate hazard ratios (HR) and 95% confidence intervals (CI) for the association between sex-specific quintiles of UPF intake (g/1000 kcal/day) and CRC incidence, overall and by anatomic location. We selected potential confounders based on the literature and observed associations between potential confounders with UPF intake and CRC in our study population: self-reported sex, race/ethnicity, education level, smoking status (incorporating time since cessation and intensity), physical activity level, standard alcohol drink equivalents per day, family history of cancer, and self-rated health status [6,7,10,28,29]. We used indicator variables to account for missing categorical data. No continuous variable had missing data, and no categorical variable had >5% missing data (S1 Table). To assess a linear trend across UPF quintiles, we used the Wald test, assigning each quintile its median value and treating it as a continuous variable. To test the proportional hazards assumption, we estimated a score test for a time-varying UPF intake [30,31]. We observed a violation of the proportional hazards assumption (*P*-value = .005). Therefore, we evaluated HR estimates for UPF intake and CRC incidence during 5-year follow-up periods, ranging from <5 to >15 years of follow-up.

Because diet quality, nutrient intake, and body mass index (BMI) could be on the causal pathway between UPF intake and incident CRC, we decided *a priori* to run separate models. In one set of analyses, we further adjusted our main model for HEI-2020 (sex-specific quartiles), calcium intake (mg/1000 kcal/day), and fiber (g/1000 kcal/day) intake and in another set, we adjusted our main model for BMI status.

In secondary analyses, we tested for effect modification, on the multiplicative scale, by diet quality, sex, and BMI status using the likelihood ratio test to compare models with and without an interaction term for UPF and the variable of interest. We present HR estimates for the joint-associations of UPF intake (sex-specific quintiles) and diet quality (>80 “good”, 51–80 “needs improvement”, and “<51 “poor”), using a common reference group, defined as the lowest quintile of UPF intake and a “good” HEI-2020 score, which we hypothesized to be the lowest risk group. We present HR estimates stratified by sex and BMI category.

We calculated HR estimates for UPF subgroups, including beverage subgroups, adapted from Martínez Steele and colleagues [24]. We also calculated mutually adjusted HR estimates for continuous intake of whole grains (servings/1000 kcal/day), dietary fiber (scaled to 10 g/1000 kcal/day), dairy (servings/1000 kcal/day), dietary calcium (scaled to 300 mg/1000 kcal/day), or meat (servings/1000 kcal/day) from UPF and non-UPF sources. Similar to the EPIC study by Kliemann and colleagues, we ran a compositional substitution model [32,33] to assess the effect of substituting 10% of grams from minimally processed food (MPF; Nova group1) for 10% of grams from UPF; this model included the relative intakes (percentage of grams) from Nova group 1, 2, and 3, and left out the relative intake from Nova group 4, such that the relative rate ratio estimate for Nova group 1 can be interpreted as substituting Nova group 1 for Nova group 4 while keeping Nova groups 2 and 3 constant. Finally, we evaluated associations for sex-specific quintiles, based on percentage energy (% kcal/day) or grams from UPF (% g/day), with incident CRC, overall and by anatomic location.

All analyses were performed using R software, version 4.4.1 (R Core Team, 2024). A two-sided *P*-value <0.05 was considered statistically significant. This study is reported as per the Strengthening the Reporting of Observational Studies in Epidemiology (STROBE) guideline (S1 STROBE Checklist). AI tools were not used to generate article contents, data, or supporting files.

## Results

Our analytic sample included 274,269 men and 187,413 women (*N* = 461,682). Median (IQR) UPF intake was 293 (198–466) g/1000 kcal/day, corresponding to 43% (36–50) of total daily energy intake. Participants included in our study tended to be younger and were more likely to be never smokers and in excellent health compared with those excluded from our analytic sample; however, UPF intake and diet quality were similar across included and excluded participants (S2 Table). With a median follow-up of 18.8 years (IQR 9.7–22.5), 10,075 CRC cases were ascertained; 4,787 were proximal colon, 2,633 were distal colon, and 2,389 were rectal cancer.

Participants in the highest quintile (Q5) of UPF intake were younger (60.7 versus 63.8 years; Tables 1 and S1) than those in the lowest quintile (Q1). They were less likely to be Asian (0.5% versus 3.0%), never smokers (32.9% versus 36.0%), or college graduates (34.4% versus 41.8%), but more likely to be alcohol nondrinkers (30.7% versus 21.5%) or have obesity (29.6% versus 15.2%). They were also less likely to exercise ≥3 times/week (40.6% versus 49.7%) or rate their health as excellent (14.2% versus 21.4%).

**Table 1:** Baseline characteristics of study participants, by sex-specific quintiles of ultra-processed food intake (g/1000 kcal/day), in the NIH-AARP Diet and Health Study (*N* = 461,682).

Characteristic, *N* (%)	UPF Q1(*n* = 92,337)	UPF Q2(*n* = 92,336)	UPF Q3(*n* = 92,336)	UPF Q4(*n* = 92,336)	UPF Q5(*n* = 92,337)	Overall (*N* = 461,682)
**Range UPF**, g/1000 kcal/day, male	3–187	>187–254	>254–350	>350–525	>525–10,043	3–10,043
**Range UPF**, g/1000 kcal/day, female	10–177	>177–241	>241–343	>343–530	>530–12,740	10–12,740
**Median UPF**, g/1000 kcal/day (IQR), male	155 (134–172)	218 (202–235)	298 (275–323)	419 (382–465)	727 (606–959)	298 (202– 465)
**Median UPF**, g/1000 kcal/day (IQR), female	147 (127–163)	207 (191–223)	285 (262–313)	418 (378–467)	757 (619–1,026)	285 (192–467)
**Median age**, years (IQR)	63.8 (59.0–67.3)	63.4 (58.5–67.0)	62.7 (57.9–66.6)	61.9 (57.3–66.1)	60.7 (56.1–65.3)	62.5 (57.7–66.6)
**Sex**, *n* (%)						
Male	54,854 (59.4)	54,854 (59.4)	54,853 (59.4)	54,854 (59.4)	54,854 (59.4)	274,269 (59.4)
Female	37,483 (40.6)	37,482 (40.6)	37,483 (40.6)	37,482 (40.6)	37,483 (40.6)	187,413 (40.6)
**Race/ethnicity**, *n* (%)						
American Indian/Alaskan Native	254 (0.3)	218 (0.2)	246 (0.3)	242 (0.3)	298 (0.3)	1,258 (0.3)
Asian	2,740 (3.0)	1,155 (1.3)	917 (1.0)	628 (0.7)	416 (0.5)	5,856 (1.3)
Hispanic	2,463 (2.7)	1988 (2.2)	1,743 (1.9)	1,423 (1.5)	1,221 (1.3)	8,838 (1.9)
Non-Hispanic Black	3,275 (3.5)	3,025 (3.3)	3,637 (3.9)	3,754 (4.1)	3,922 (4.2)	17,613 (3.8)
Non-Hispanic White	81,869 (88.7)	84,657 (91.7)	84,634 (91.7)	85,149 (92.2)	85,221 (92.3)	421,530 (91.3)
Pacific Islander	200 (0.2)	104 (0.1)	104 (0.1)	80 (0.1)	64 (0.1)	552 (0.1)
Unknown	1,536 (1.7)	1,189 (1.3)	1,055 (1.1)	1,060 (1.1)	1,195 (1.3)	6,035 (1.3)
**Never smoker**, *n* (%)	33,247 (36.0)	33,732 (36.5)	33,820 (36.6)	33,245 (36.0)	30,420 (32.9)	164,464 (35.6)
**College graduate**, *n* (%)	38,610 (41.8)	38,487 (41.7)	36,425 (39.4)	34,301 (37.1)	31,702 (34.3)	179,525 (38.9)
**Exercises ≥3 times a week**, *n* (%)	45,863 (49.7)	44,994 (48.7)	43,444 (47.1)	41,456 (44.9)	38,447 (41.6)	214,204 (46.4)
**Alcohol nondrinkers**, *n* (%)	19,819 (21.5)	19,019 (20.6)	20,243 (21.9)	22,589 (24.5)	28,362 (30.7)	110,032 (23.8)
**BMI ≥ 30 kg/m**^**2**^, *n* (%)	14,045 (15.%)	15,948 (17.3)	18,784 (20.3)	21,773 (23.6)	27,328 (29.6)	97,878 (21.2)
**Excellent self-rated health**, *n* (%)	19,804 (21.4)	17,524 (19.0)	15,975 (17.3)	14,617 (15.8)	13,112 (14.2)	81,032 (17.6)
**Family history of cancer**, *n* (%)	44,444 (48.1)	45,407 (49.2)	45,069 (48.8)	44,931 (48.7)	44,322 (48.0)	224,173 (48.6)
**Median energy intake,** kcal/day (IQR)	1,547 (1,165-2025)	1,622 (1,241-2,106)	1,671 (1,268–2,172)	1,615 (1,246–2,126)	1,520 (1,126–2020)	1,600 (1,208–2,091)
**Median HEI-2020 score** (IQR)^a^	70.9 (64.1–76.7)	69.9 (63.0–75.7)	68.9 (62.1–74.7)	67.6 (60.6–73.6)	65.6 (58.3–72.0)	68.6 (61.5–74.7)
**Median dietary calcium,** mg/1000 kcal/day (IQR)	417 (327–555)	419 (335–542)	416 (332–535)	403 (321–519)	405 (315–528)	412 (326–536)
**Median dietary fiber,** g/1000 kcal/day (IQR)	11.8 (9.3–14.9)	11.4 (9.2–14.0)	10.8 (8.8–13.4)	10.4 (8.4–12.9)	10.0 (7.8–12.6)	10.9 (8.7–13.6)

^a^Total HEI-2020 score (out of 100 points) measures alignment with the 2020−2025 Dietary Guidelines for Americans; components and scoring standards are the same as the HEI-2015.

Abbreviations: BMI, body mass index; HEI-2020, Healthy Eating Index-2020; IQR, interquartile range; Q1, first quintile; Q2, second quintile; Q3, third quintile; Q4, fourth quintile; Q5, fifth quintile; UPF, ultra-processed food.

In multivariable-adjusted models, using age as the time metric, for the full follow-up period, no associations were observed between UPF (g/1000 kcal/day) quintiles and relative CRC incidence overall (HR_Q5vs.Q1_ 0.97; 95% CI [0.91,1.03]; *P*_trend_ = 0.55) or by subsite (proximal colon: HR_Q5vs.Q1_ 1.02; 95% CI [0.93,1.12]; *P*_trend_ = 0.20; distal colon: HR_Q5vs.Q1_ 0.94; 95% CI [0.83,1.06]; *P*_trend_ = 0.64; rectum: HR_Q5vs.Q1_ 0.91; 95% CI [0.80,1.03]; *P*_trend_ = 0.93; Table 2). Adjusting for HEI-2020 score and nutrient intake (HR_Q5vs.Q1_ 0.94; 95% CI [0.88,1.00]; *P*_trend_ = 0.69) or BMI status (HR_Q5vs.Q1_ = 0.94; 95% CI [0.89,1.01]; *P*_trend_ = 0.86) did not appreciably alter HR estimates (<10% change).

**Table 2:** Association of ultra-processed food intake (sex-specific quintiles of nutrient-adjusted g/1000 kcal/day) with colorectal cancer incidence overall and by anatomic location^a^ in the NIH-AARP Diet and Health Study (*N* = 461,682).

	UPF Q1 (*n* = 92,337)	UPF Q2 (*n* = 92,336)	UPF Q3 (*n* = 92,336)	UPF Q4 (*n* = 92,336)	UPF Q5 (*n* = 92,337)	*P*-trend^b^
**Colorectal cancer**						
No. of cases	2,152	1946	1917	2031	2029	
Base model, HR (95% CI)^c^	1.00 (ref)	0.90 (0.84,0.95)	0.89 (0.84,0.95)	0.96 (0.90,1.02)	1.01 (0.95,1.07)	0.02
Multivariable model, HR (95% CI)^d^	1.00 (ref)	0.91 (0.85,0.96)	0.90 (0.84,0.95)	0.95 (0.89,1.01)	0.97 (0.91,1.03)	0.55
+ HEI and dietary factors, HR (95% CI)^e^	1.00 (ref)	0.90 (0.85,0.96)	0.89 (0.84,0.95)	0.93 (0.88,0.99)	0.94 (0.88,1.00)	0.69
+ BMI, HR (95% CI)^f^	1.00 (ref)	0.90 (0.85,0.96)	0.89 (0.84,0.95)	0.94 (0.88,1.00)	0.94 (0.89,1.01)	0.86
**Distal colon cancer**						
No. of cases	555	500	525	522	531	
Base model, HR (95% CI)^c^	1.00 (ref)	0.89 (0.79,1.00)	0.93 (0.83,1.05)	0.93 (0.83,1.05)	0.98 (0.87,1.11)	0.58
Multivariable model, HR (95% CI)^d^	1.00 (ref)	0.90 (0.80,1.02)	0.94 (0.84,1.06)	0.93 (0.82,1.05)	0.94 (0.83,1.06)	0.64
+ HEI and dietary factors, HR (95% CI)^e^	1.00 (ref)	0.89 (0.79,1.01)	0.92 (0.82,1.04)	0.90 (0.79,1.01)	0.90 (0.79,1.01)	0.23
+ BMI, HR (95% CI)^f^	1.00 (ref)	0.90 (0.79,1.01)	0.93 (0.82,1.05)	0.90 (0.80,1.02)	0.90 (0.80,1.02)	0.26
**Proximal colon cancer**						
No. of cases	994	972	881	979	961	
Base model, HR (95% CI)^c^	1.00 (ref)	0.98 (0.89,1.07)	0.90 (0.82,0.99)	1.02 (0.94,1.12)	1.07 (0.98,1.17)	0.01
Multivariable model, HR (95% CI)^d^	1.00 (ref)	0.97 (0.89,1.06)	0.89 (0.82,0.98)	1.00 (0.92,1.10)	1.02 (0.93,1.12)	0.20
+ HEI and dietary factors, HR (95% CI)^e^	1.00 (ref)	0.97 (0.89,1.06)	0.89 (0.81,0.98)	0.99 (0.91,1.08)	1.00 (0.91,1.10)	0.45
+ BMI, HR (95% CI)^f^	1.00 (ref)	0.97 (0.89,1.06)	0.89 (0.81,0.97)	0.99 (0.91,1.08)	1.00 (0.91,1.09)	0.45
**Rectal cancer**						
No. of cases	535	424	473	478	479	
Base model, HR (95% CI)^c^	1.00 (ref)	0.78 (0.69,0.89)	0.87 (0.77,0.99)	0.89 (0.78,1.00)	0.92 (0.81,1.04)	0.83
Multivariable model, HR (95% CI)^d^	1.00 (ref)	0.80 (0.70,0.91)	0.90 (0.79,1.01)	0.90 (0.80,1.02)	0.91 (0.80,1.03)	0.93
+ HEI and dietary factors, HR (95% CI)^e^	1.00 (ref)	0.80 (0.70,0.90)	0.89 (0.78,1.00)	0.88 (0.78,1.00)	0.88 (0.77,1.00)	0.48
+ BMI, HR (95% CI)^f^	1.00 (ref)	0.80 (0.70,0.91)	0.90 (0.79,1.01)	0.90 (0.79,1.02)	0.91 (0.80,1.03)	0.85

^a^Site codes C188, C189, and C260 are included in the CRC analysis of 10,075 cases but censored at diagnosis date in subsite analyses.

^b^*P*-trend based on the Wald test, assigning each UPF quintile to its median value and treating it as a continuous variable in the Cox proportional hazards regression model.

^c^Adjusted for age in years (underlying time metric), sex (male/female), and total daily energy (kcal/day) for nutrient density adjustment.

^d^Adjusted for age in years (underlying time metric), total daily energy (kcal/day), sex (male, female), race/ethnicity (American Indian/Alaskan Native, Asian, Hispanic, Non-Hispanic Black, Non-Hispanic White, Pacific Islander, unknown), and smoking status (never, former, current, unknown) by intensity (cigarettes per day: 1–10, 11–20, 21–30, 31–40, 41–60, 61+) and time since cessation (10+ , 5–9, 1–4 years ago, within the last year), education level (11 years or less; 12 years, completed high school, or GED; post-high school training; some college; college and post graduate; unknown), physical activity level (never/rarely, low, moderate, high, unknown), alcohol intake (0, <1, 1–2, 3–4, 5+ standard drinks/day), family history of cancer (yes, no, unknown), and self-reported health status (excellent, very good, good, fair).

^e^Multivariable model further adjusted for Healthy Eating Index (HEI)-2020 score (males - quartile 1: 21.5 to <60.6, quartile 2: 60.6 to <67.7, quartile 3: 67.7 to <74.0, quartile 4: 74.0 to 97.5; females - quartile 1: 25.9 to <63.0, quartile 2: 63.0 to <69.8, quartile 3: 69.8 to <75.6, quartile 4: 75.6 to 95.8), dietary calcium (males - quintile 1: 79.2 to <303.6, quintile 2: 303.6 to <367.0, quintile 3: 367.0 to <438.5, quintile 4: 438.5 to <551.3, quintile 5: 551.3 to 2312.8 mg/1000 kcal/day; females - quintile 1: 53.9 to <318.1, quintile 2: 318.1 to <392.6, quintile 3: 392.6 to <476.6, quintile 4: 476.6 to <605.2, quintile 5: 605.2 to 2321.7 mg/1000 kcal/day), and dietary fiber (males - quintile 1: 0.56 to <7.93, quintile 2: 7.93 to <9.69, quintile 3: 9.69 to <11.44, quintile 4: 11.44 to <13.89, quintile 5: 13.89 to 51.98 g/1000 kcal/day; females - quintile 1: 1.01 to <8.57, quintile 2: 8.57 to <10.47, quintile 3: 10.47 to <12.34, quintile 4: 12.34 to <14.93, quintile 5: 14.93 to 57.17 g/1000 kcal/day).

^f^Multivariable model, not including HEI and dietary factors, further adjusted for BMI category (<18.5, 18.5 to <25, 25 to <30, ≥30 kg/m^2^, unknown).

Abbreviations: BMI, body mass index; CI, confidence interval; GED, General Equivalency Diploma; HEI, Healthy Eating Index 2020; HR, hazard ratio first quintile; Q2, second quintile; Q3, third quintile; Q4, fourth quintile; Q5, fifth quintile; UPF, ultra-processed food.

In estimating the joint effect of UPF intake and diet quality, no statistical evidence of a multiplicative interaction (*P*_heterogeneity_ = 0.11) was observed. HR estimates across joint UPF-HEI categories were inconsistent; compared to being in the lowest quintile of UPF intake with good diet quality, being in the 2nd (HR 1.28; 95% CI [1.01,1.50]) or 5th (HR 1.22; 95% CI [1.01,1.48]) quintile with poor diet quality was associated with higher CRC incidence (Fig 1). No evidence of effect modification by sex (*P*_heterogeneity_ = 0.30; S3, S4 Tables) or BMI status (*P*_heterogeneity_ = 0.38; S5 Table) was observed. HR estimates during 5-year follow-up periods were generally similar (Table 3).

**Table 3:** Association of ultra-processed food intake (sex-specific quintiles of nutrient-adjusted g/1000 kcal/day) with colorectal cancer incidence according to follow-up time (1to <5 y, 5 to <10 y, 10 to <15 y, ≥15 y) in the NIH-AARP Diet and Health Study (*N* = 461,682).

Follow up period	UPF Q1	UPF Q2	UPF Q3	UPF Q4	UPF Q5	*P*-trend ^a^
Overall						
No. of participants	92,337	92,336	92,336	92,336	92,337	
No. of cases	2,152	1946	1917	2031	2029	
Base model, HR (95% CI)^b^	1.00 (ref)	0.90 (0.84,0.95)	0.89 (0.84,0.95)	0.96 (0.90,1.02)	1.01 (0.95,1.07)	0.02
Multivariable model, HR (95% CI) ^c^	1.00 (ref)	0.91 (0.85,0.96)	0.90 (0.84,0.95)	0.95 (0.89,1.01)	0.97 (0.91,1.03)	0.55
1 to <5 years						
No. of participants	92,337	92,336	92,336	92,336	92,337	
No. of cases	544	464	477	516	490	
Base model, HR (95% CI)^b^	1.00 (ref)	0.87 (0.77,0.99)	0.94 (0.83,1.06)	1.06 (0.94,1.19)	1.09 (0.96,1.23)	0.005
Multivariable model, HR (95% CI) ^c^	1.00 (ref)	0.88 (0.78,1.00)	0.94 (0.83,1.07)	1.05 (0.93,1.19)	1.05 (0.93,1.19)	0.04
5 to <10 years						
No. of participants	81,689	82,134	82,236	82,685	82,366	
No. of cases	645	563	541	569	612	
Base model, HR (95% CI)^b^	1.00 (ref)	0.87 (0.78,0.99)	0.86 (0.77,0.97)	0.93 (0.83,1.04)	1.07 (0.96,1.20)	.008
Multivariable model, HR (95% CI) ^c^	1.00 (ref)	0.89 (0.79,0.99)	0.87 (0.78,0.98)	0.93 (0.83,1.05)	1.04 (0.93,1.17)	0.05
10 to <15 years						
No. of participants	67,500	68,496	68,737	69,290	68,383	
No. of cases	482	439	474	458	465	
Base model, HR (95% CI) ^b^	1.00 (ref)	0.89 (0.78,1.02)	0.98 (0.86,1.11)	0.96 (0.84,1.09)	1.04 (0.92,1.19)	0.15
Multivariable model, HR (95% CI) ^c^	1.00 (ref)	0.91 (0.80,1.03)	0.99 (0.87,1.13)	0.96 (0.84,1.09)	1.00 (0.88,1.14)	0.51
15+ years						
No. of participants	55,074	56,311	56,756	57,133	56,068	
No. of cases	481	480	425	488	462	
Base model, HR (95% CI) ^b^	1.00 (ref)	0.97 (0.86,1.11)	0.87 (0.76,0.99)	1.01 (0.89,1.15)	1.03 (0.91,1.17)	0.26
Multivariable model, HR (95% CI) ^c^	1.00 (ref)	0.98 (0.87,1.12)	0.87 (0.76,0.99)	1.00 (0.88,1.14)	0.99 (0.87,1.13)	0.67

^a^*P*-trend based on the Wald test, treating UPF intake as a continuous variable by assigning each quintile to its median value in the Cox proportional hazards regression model.

^b^Adjusted for age in years (underlying time metric), sex (male/female), and total daily energy (kcal/day) for nutrient density adjustment.

^c^Adjusted for age in years (underlying time metric), total daily energy (kcal/day), sex (male, female), race/ethnicity (American Indian/Alaskan Native, Asian, Hispanic, Non-Hispanic Black, Non-Hispanic White, Pacific Islander, unknown), smoking status (never, former, current, unknown) by intensity (cigarettes per day: 1–10, 11–20, 21–30, 31–40, 41–60, 61+) and time since cessation (10+ , 5–9, 1–4 years ago, within the last year), education level (11 years or less; 12 years, completed high school, or GED; post-high school training; some college; college and post graduate; unknown), physical activity level (never/rarely, low, moderate, high, unknown), alcohol intake (0, < 1, 1–2, 3–4, 5+ standard drinks/day), family history of cancer (yes, no, unknown), and self-reported health status (excellent, very good, good, fair, unknown).

Abbreviations: CI, confidence interval; GED, General Equivalency Diploma; HR, hazard ratio; first quintile; Q2, second quintile; Q3, third quintile; Q4, fourth quintile; Q5, fifth quintile; UPF, ultra-processed food.

**Fig 1:**
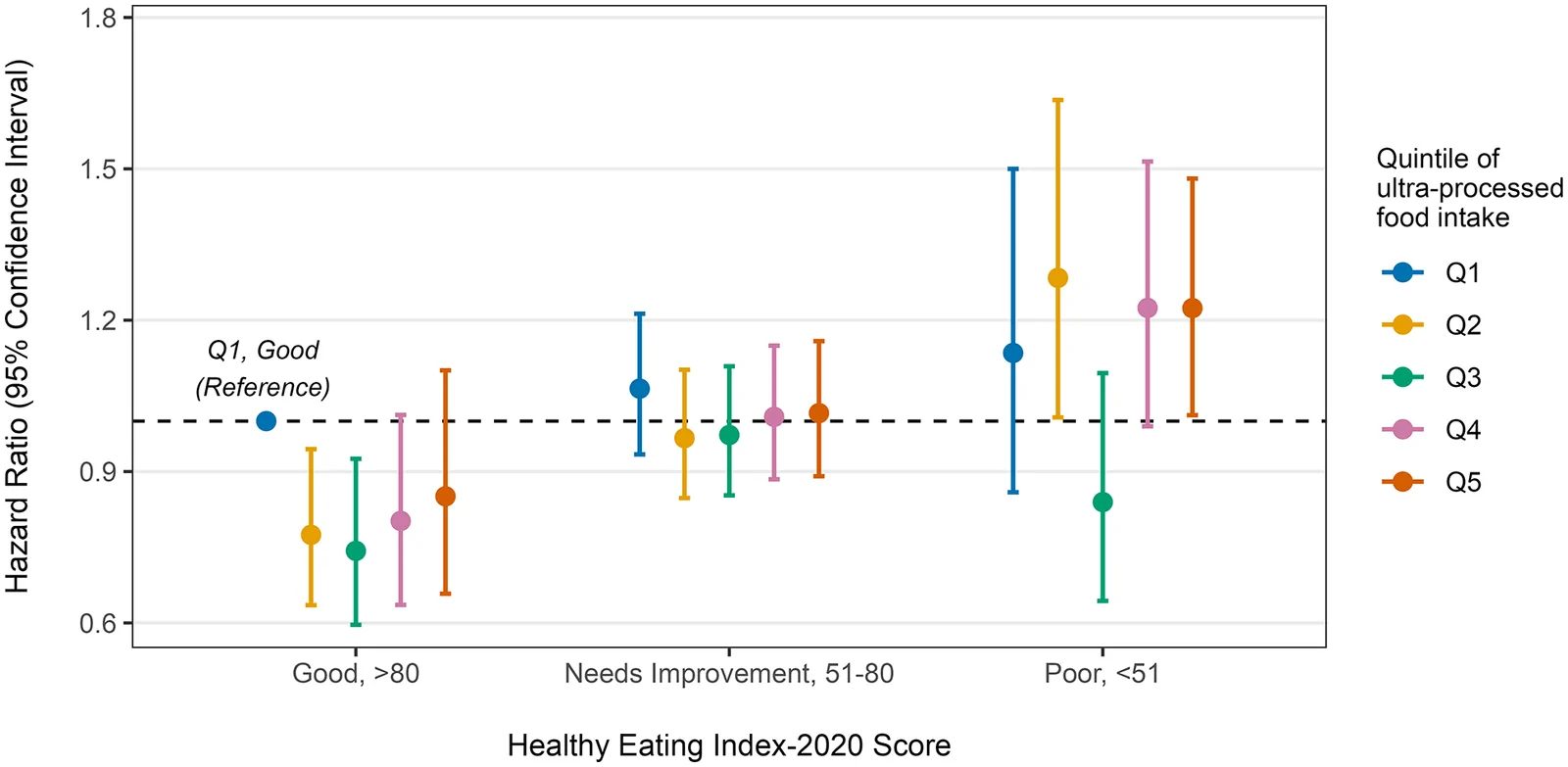
Associations for joint effect of ultra-processed food intake (g/1000 kcal/day), defined using sex-specific quintiles, and diet quality, measured using the Healthy Eating Index-2020, with colorectal cancer incidence in the NIH-AARP Diet and Health Study cohort (*N* = 461,682). ^a^Hazard ratios and 95% confidence intervals are estimated using a Cox proportional hazard regression model adjusted for age in years (underlying time metric), total daily energy (kcal/day), sex (male, female), race/ethnicity (American Indian/Alaskan Native, Asian, Hispanic, Non-Hispanic Black, Non-Hispanic White, Pacific Islander, unknown), smoking status (never, former, current, unknown) by intensity (cigarettes per day: 1–10, 11–20, 21–30, 31–40, 41–60, 61+) and time since cessation (10 + , 5–9, 1–4 years ago, within the last year), education level (11 years or less; 12 years, completed high school, or GED; post-high school training; some college; college and post graduate; unknown), physical activity level (never/rarely, low, moderate, high, unknown), alcohol intake (0, < 1, 1–2, 3–4, 5+ standard drinks/day), family history of cancer (yes, no, unknown), and self-reported health status (excellent, very good, good, fair). UPF Q1 and HEI-2020 score >80 serves as the common reference group. Likelihood ratio test comparing models with and without UPF*HEI-2020 interaction term: *P* = 0.11. Abbreviations: CI, confidence interval; GED, General Equivalency Diploma; HEI, Healthy Eating Index; HR, hazard ratio; first quintile; Q2, second quintile; Q3, third quintile; Q4, fourth quintile; Q5, fifth quintile; UPF, ultra-processed food.

In the UPF subgroup analysis, we observed inverse associations for higher intake of UPF that are major sources of dietary calcium or dairy and dietary fiber or whole grains with CRC incidence. These subgroups included bread (HR_Q5vs.Q1_ 0.91; 95% CI [0.86,0.97]; *P*_trend_ < 0.001), frozen desserts and milk drinks (HR_Q5vs.Q1_ 0.82; 95% CI [0.77,0.88]; *P*_trend_ < 0.001), and breakfast cereals (HR_Q5vs.Q1_ 0.84; 95% CI [0.79,0.90]; *P*_trend_ < 0.001; S1 Fig). Conversely, higher intake of reconstituted meat and fish products was positively associated with CRC incidence (HR_Q5vs.Q1_ 1.10; 95% CI [1.04,1.18]; *P*_trend_ < 0.001).

In our analysis, 82.8%, 33.5%, 31.7%, and 23.5% of whole grain, dietary fiber, dietary calcium, and dairy intake, respectively, as well as 74.3% of processed meat intake came from UPF sources (S6 Table). UPF and non-UPF intakes of CRC-related dietary components were moderately correlated, with correlations between 0.37 for whole grain and 0.48 for meat. In mutually-adjusted models, intakes of dietary calcium from UPF sources (HR_300 mg/day_ 0.77; 95% CI [0.69,0.86]) and non-UPF sources (HR_300 mg/day_ 0.90, 95% CI [0.87,0.93]) as well as dairy from UPF sources (HR _1 serving/day_ 0.75, 95% CI [0.62,0.91]) and non-UPF sources (HR_1 serving/day_ 0.90; 95% CI [0.87,0.94]) were independently associated with lower CRC incidence, and intakes of meat from UPF sources (HR_1 serving/day_ 1.08; 95% CI [1.02,1.14]) and non-UPF sources (HR_1 serving/day_ 1.04; 95% CI [1.01,1.06]) were independently associated with higher CRC incidence. Whole grain (HR_1 serving/day_ 0.87; 95% CI [0.82,0.92]) and fiber (HR_10 gram/day_ 0.72; 95% CI [0.64,0.80]) intakes from UPF were inversely associated with CRC incidence but their non-UPF counterparts in the model were not (Fig 2).

**Fig 2:**
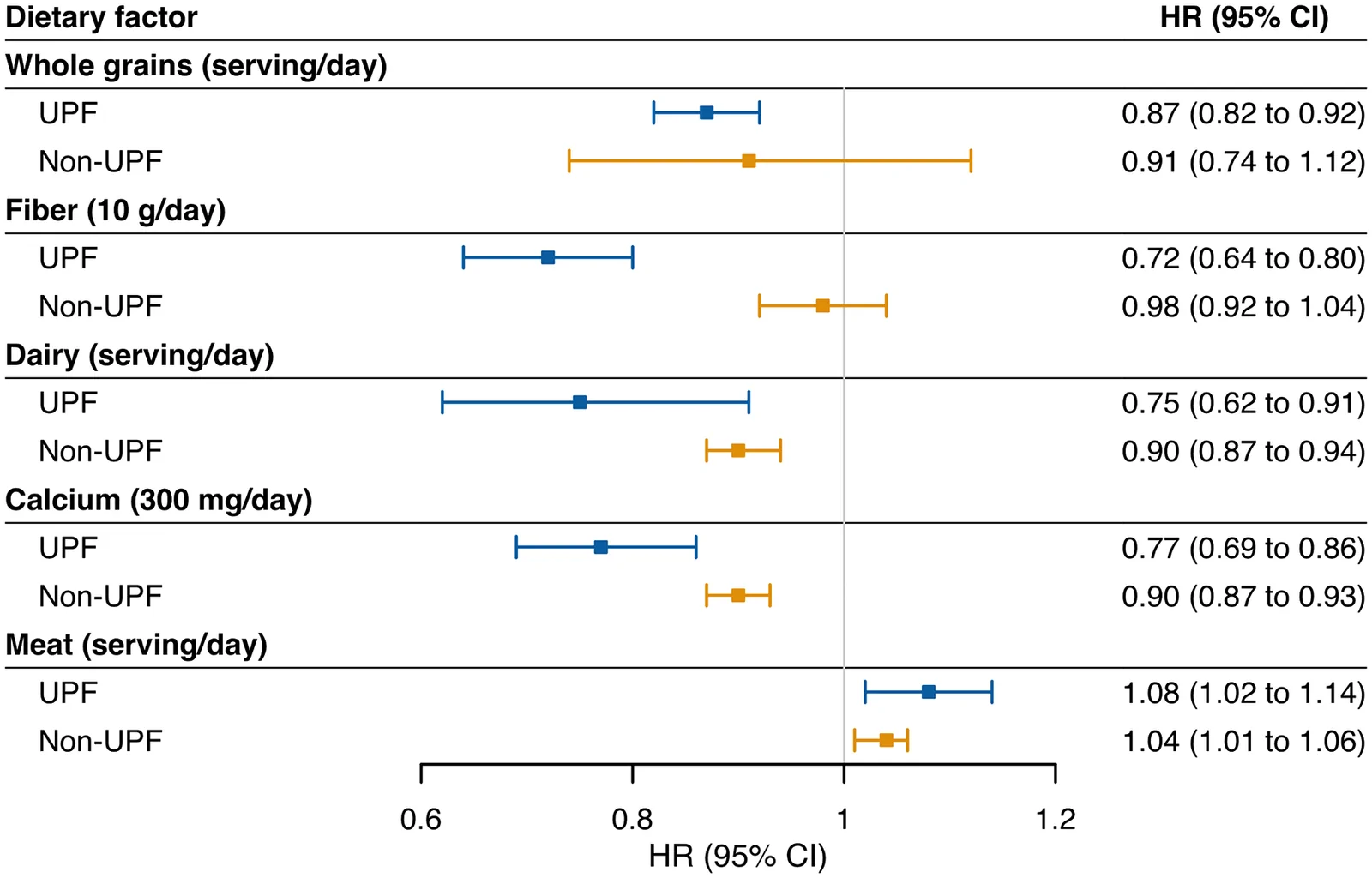
Mutually adjusted associations for select dietary factors with colorectal cancer incidence by Nova classification of food source (UPF or non-UPF) in the NIH-AARP Diet and Health Study (*N* = 461,682). Hazard ratios and 95% confidence intervals are estimated using a Cox proportional hazard regression model adjusted for age in years (underlying time metric), total daily energy (kcal/day), sex (male, female), race/ethnicity (American Indian/Alaskan Native, Asian, Hispanic, Non-Hispanic Black, Non-Hispanic White, Pacific Islander, unknown), smoking status (never, former, current, unknown) by intensity (cigarettes per day: 1–10, 11–20, 21–30, 31–40, 41–60, 61+) and time since cessation (10+ , 5–9, 1–4 years ago, within the last year), education level (11 years or less; 12 years, completed high school, or GED; post-high school training; some college; college and post graduate; unknown), physical activity level (never/rarely, low, moderate, high, unknown), alcohol intake (0, < 1, 1–2, 3–4, 5+ standard drinks/day), family history of cancer (yes, no, unknown), and self-reported health status (excellent, very good, good, fair). The model accounts for mutual adjustments between sources from both non-UPF (Nova 1–3) and UPF (Nova 4). Abbreviations: CI, confidence interval; GED, General Equivalency Diploma; HR, hazard ratio; UPF, ultra-processed food.

Following multivariable adjustment (main model), substituting 10% of grams from UPF with 10% from MPF was associated with a 2% lower incidence of CRC (HR 0.98; 95% CI [0.96,0.99]). UPF intake, defined using percentage energy or grams (S7, S8 Tables), generally yielded similar null HR estimates. In contrast, those in the highest, compared to the lowest, quintile of percentage energy from UPF had lower rectal cancer incidence (HR_Q5vs.Q1_ 0.86; 95% CI [0.76,0.98]; *P*_trend_ = 0.05); adjusting for diet quality, fiber intake, and calcium intake strengthened the inverse association (HR_Q5vs.Q1_ 0.76; 95% CI [0.67,0.88]; *P*_trend_ < 0.001).

## Discussion

In the NIH-AARP Diet and Health Study, with an analytic cohort of 461,682 US adults who were followed for cancer for more than 20 years, we found little evidence of an association between total UPF intake and CRC incidence. Interestingly, we found that UPF contributed to intake of dietary factors that have previously been associated with lower CRC incidence in NIH-AARP [5,34–36], namely whole grain (82.8% from UPF), dietary fiber (33.5%), dairy (23.5%), and dietary calcium (31.7%). UPF also contributed to intake of processed meat (74.3% from UPF), which is an established risk factor for CRC [37]. Finally, we found that HR estimates for intake of whole grain, dietary fiber, dairy, calcium, and processed meat reflected current dietary guidance for CRC prevention [5].

An umbrella review [38], which synthesized results from epidemiologic studies [6,7,10,39–41], concluded that the quality of evidence for an association between UPF and CRC was very low [38]. Prospective studies on UPF intake and CRC incidence have been mixed. In the French NutriNet-Santé cohort, UPF intake (% grams/day) was associated with higher CRC incidence; however, the association, which was based on 153 CRC cases, was not statistically significant [7]. Similarly, analyses of multiple cancer types in the UK Biobank (UKB) and European Prospective Investigation into Cancer (EPIC) found no association between UPF intake (% grams/day) and CRC incidence [10,33]. An analysis of data from three US cohorts of health professionals found that higher UPF intake (servings/day) was associated with higher CRC incidence in men, but not in women [6], whereas, a subsequent analysis of data from the EPIC study showed a 6% higher CRC incidence for each 10% increase in UPF intake (% g/day) in women but not in men [9]. In EPIC, Al Nahas and colleagues also found that substituting 10% of grams from UPF with 10% from MPF was associated with a 6% lower hazard of CRC (HR = 0.94, 0.90,0.97) [9]. We conducted a similar analysis and found that in NIH-AARP the same substitution was associated with a 2% lower hazard of CRC (HR = 0.98, 0.96,0.99).

One plausible explanation for varying results is that the composition of diets high in UPF intake differs across populations. For example, higher intake of ultra-processed meat products (e.g., mean 36.2 g/d in EPIC versus 20.1 g/d in NIH-AARP; S8 Table) and lower intake of some ultra-processed breakfast cereals (e.g., mean 5.0 g/d in EPIC versus 15.2 g/d in NIH-AARP) and breads (e.g., mean 43.6 g/d in EPIC versus 57.3 g/d in NIH-AARP) [33], which serve a sources of whole grain intake, could contribute to observed differences across studies or across strata (e.g., sex) within a study and explain why the HR estimate for replacing 10% grams from UPF with MPF was similar in direction to the EPIC analysis but weaker in magnitude in NIH-AARP. Compositional differences may also contribute to inconsistent findings at the UPF subgroup level. For example, average intake of added sugar or fiber from breakfast cereal may vary substantially from one population to another. Other studies of older, well-educated US adults have reported inverse associations for certain UPF subgroups, such as cereal intake with type 2 diabetes incidence [18] and yogurt and dairy based desserts with CRC incidence [6], that reflect UPF subgroup associations observed herein, but the generalizability of these findings may be limited. Other plausible explanations include differences in type and frequency of dietary assessment, length of follow-up for cancer incidence, and confounder adjustment.

Poor diet quality has been associated with higher CRC incidence in the NIH-AARP Study [42–44]. Herein, we found that poor diet quality was associated with an elevated CRC hazard in both lower and higher quintiles of UPF intake. Nutrient-based and food-processing-based frameworks, such as Nova, capture distinct dimensions of diet and should not be assumed to be interchangeable but can be examined in combination. Looking specifically at nutrient and food group intake, we found that dairy and calcium intake were associated with lower CRC incidence, but meat intake was associated with higher CRC incidence in models containing intake estimates (e.g., calcium) from both from UPF and non-UPF. In contrast, we observed inverse associations for whole grain and fiber intake from UPF, but not non-UPF, sources. This could be explained by the fact that <20% of estimated whole grain intake came from non-UPF sources, indicating that these foods were consumed by fewer people or in small amounts and likely resulting in less precise HR estimates. For fiber, although most intake came from non-UPF sources, the association with lower CRC incidence in NIH-AARP is for fiber from whole grains [34], which come mostly from UPF sources in our study, likely explaining the nonsignificant inverse association for non-UPF fiber.

Our study has limitations. First, the FFQ and databases that our study relied on were designed to measure nutrient intake not to assess food processing level. Individuals who reported eating yogurt, for example, could have consumed flavored or plain yogurt, which fall under different Nova groups, but this level of granularity was not captured directly. Therefore, estimates derived from linkage to US population level food consumption data were applied equally to all participants who consume a given food item, which could limit the exposure differences that can be captured between individuals and potentially introduce bias. Still, we previously demonstrated that the NIH-AARP FFQ performs reasonably well, as compared with two 24 h dietary recalls, for estimating intake according to the Nova system [23] with attenuation factors comparable to those for nutrients and food groups [20]. Exposure misclassification may have been compounded in food group and nutrient analyses since intake estimation relied on an additional level of classification; thus, these analyses should be viewed as exploratory and interpreted with caution. Participants were older, mostly white, US adults who were recruited in the mid-1990s, and UPF intake was measured only once at baseline. Consequently, our findings may not generalize to younger more diverse populations or reflect the everchanging US food supply. Given substantial changes in UPF availability and consumption over the past few decades [45], our estimates likely underestimate current UPF intake levels, which may also contribute to observed null findings. The single baseline assessment of dietary intake, combined with the large sample size, and extended follow-up time may have also contributed to the statistically significant departure from proportionality despite generally consistent HR estimates across follow-up periods. Limited variation across quintiles of UPF intake may also contribute to our null findings. UPF often contain food additives that may negatively impact gut health [46–48]. For example, emulsifiers have been found to disrupt the intestinal mucosal barrier, leading to dysbiosis, increased gut permeability, and low-grade inflammation—factors linked to metabolic syndrome and CRC [49–51]. However, we were unable to estimate associations with food additives due to the absence of ingredient level data. Residual confounding by unmeasured (e.g., use of nonsteroidal anti-inflammatory drugs) or poorly measured (e.g., socioeconomic status) confounders is possible. We estimate that an unmeasured confounder would need to be associated with more than a 2.1-fold higher relative rate of UPF exposure and CRC conditional on measured covariates, to fully explain away the strongest observed association, which was for fiber (i.e., HR = 0.72), in our study [52]. Finally, it is important to note that while secondary analyses were hypothesis driven and nested within the main analysis, statistically significant findings should be interpreted with caution owing to multiple testing and the potential for type I error.

Strengths of our study include its prospective design, large size, and extended follow-up for cancer outcomes, which afforded sufficient case numbers to evaluate associations by tumor location and diet quality. Furthermore, our method for disaggregating FFQ items to assign Nova classification [23] not only enhances reliability of our UPF measure but also allowed us to estimate intake of nutrients and food groups by Nova classification of the food source.

In conclusion, we found that UPF intake was not associated with CRC incidence in a large cohort of older US adults. Understanding how current evidence-based dietary guidance for CRC prevention intersects with research on food processing is critical. Our results support a role for whole grain, fiber, dairy and calcium intake in CRC prevention that was not diminished by food processing level. However, poor diet quality is a risk factor for CRC [42–44,53] and dietary patterns that limit intake of added sugars, sodium, saturated fat, and refined grains and prioritize whole foods lower the relative risk of chronic disease-related mortality [54]. In addition, substituting 10% of grams from UPF with 10% from MPF was associated with a 2% lower incidence of CRC. Future studies are needed to replicate and extend our findings, and novel methods for measuring UPF and additive intake are needed to elucidate what aspects of UPF are associated with CRC development and progression.

## Supporting information

S1 Table Baseline characteristics of the study participants, by nutrient-adjusted ultra-processed food (UPF) intake (*N* = 461,682). (DOCX)

S2 Table Baseline characteristics of study participants who were in the NIH-AARP Diet and Health Study by inclusion in the analytic sample. (DOCX)

S3 Table Association of ultra-processed food intake (quintiles of nutrient-adjusted g/1000 kcal/day) with colorectal cancer incidence by anatomic location in male participants in the NIH-AARP Diet and Health Study (*N* = 274,269). (DOCX)

S4 Table Association of ultra-processed food intake (quintiles of nutrient-adjusted g/1000 kcal/day) with colorectal cancer incidence by anatomic location in female participants in the NIH-AARP Diet and Health Study (*N* = 187,413). (DOCX)

S5 Table Association of ultra-processed food intake (quintiles of nutrient-adjusted g/1000 kcal/day) with colorectal cancer incidence stratified by BMI category. (DOCX)

S6 Table Dietary calcium, dairy, whole grain, fiber, and meat intake by Nova group in the NIH-AARP Diet and Health Study (*N* = 461,682). (DOCX)

S7 Table Association of UPF intake (sex-specific quintiles of % kcal/day) with colorectal cancer incidence in the NIH-AARP Diet and Health Study (*N* = 461,682). (DOCX)

S8 Table Association of UPF intake (sex-specific quintiles of % grams/day) with colorectal cancer incidence in the NIH-AARP Diet and Health Study (*N* = 461,682). (DOCX)

S9 Table Absolute intakes and relative contributions of subgroups to UPF intake (Nova group 4) in NIH-AARP (*N* = 461,682). (DOCX)

S1 Fig Intake of ultra-processed food subgroups and colorectal cancer incidence in the NIH-AARP Diet and Health Study (*N* = 461,682). (DOCX)

S1 Checklist STROBE Statement—Checklist of items that should be included in reports of cohort studies. (DOCX)

## Abbreviations

BMI: body mass index
CI: confidence intervals
CRC: colorectal cancer
CSFII: Continuing Survey of Food Intake of Individuals
EPIC: European Prospective Investigation into Cancer
FFQ: food frequency questionnaire
FNDDS: Food and Nutrient Database for Dietary Studies
FP: Food Patterns
FPID: Food Patterns Ingredient Database
HEI: Healthy Eating Index
HR: hazard ratios
IMS: Information Management Services
IQR: interquartile range
NIH: National Institutes of Health
SR: standard reference
UKB: UK Biobank
UPF: ultra-processed food
