# Supplementary material for: Associations of food processing and dietary risk factors with colorectal cancer incidence in the NIH-AARP Diet and Health Study: A cohort study
Source: PLoS Med. 2026 Oct 15;23(10):e1005251. doi: 10.1371/journal.pmed.1005251 (PMC13671506; doi:10.1371/journal.pmed.1005251)
Supplement: Supplementary file 1 [file pmed.1005251.s001.docx]

**S1 Table**. Baseline characteristics of the study participants, by nutrient-adjusted ultra-processed food (UPF) intake^a^ (N=461,682)

| **Characteristic, N (%)**^b^ | **Quintile 1 (N=92337)** | **Quintile 2 (N=92336)** | **Quintile 3 (N=92336)** | **Quintile 4 (N=92336)** | **Quintile 5 (N=92337)** |
| --- | --- | --- | --- | --- | --- |
| **Median grams of UPF**, g/day (IQR) | 226 (162-308) | 346 (261-456) | 491 (364-649) | 672 (524-905) | 1160 (889-1787) |
| **Median grams of UPF**, g/1000 kcal/day (IQR) | 152 (131-168) | 213 (198-230) | 293 (270-319) | 419 (380-466) | 738 (611-986) |
| **Median age**, years (IQR) | 63.8 (59.0-67.3) | 63.4 (58.5-67.0) | 62.7 (57.9-66.6) | 61.9 (57.3-66.1) | 60.7 (56.1-65.3) |
| **Sex** |  |  |  |  |  |
| Male | 54854 (59.4%) | 54854 (59.4%) | 54853 (59.4%) | 54854 (59.4%) | 54854 (59.4%) |
| Female | 37483 (40.6%) | 37482 (40.6%) | 37483 (40.6%) | 37482 (40.6%) | 37483 (40.6%) |
| **Race/ethnicity** |  |  |  |  |  |
| American Indian/Alaskan Native | 254 (0.3%) | 218 (0.2%) | 246 (0.3%) | 242 (0.3%) | 298 (0.3%) |
| Asian | 2740 (3.0%) | 1155 (1.3%) | 917 (1.0%) | 628 (0.7%) | 416 (0.5%) |
| Hispanic | 2463 (2.7%) | 1988 (2.2%) | 1743 (1.9%) | 1423 (1.5%) | 1221 (1.3%) |
| Non-Hispanic Black | 3275 (3.5%) | 3025 (3.3%) | 3637 (3.9%) | 3754 (4.1%) | 3922 (4.2%) |
| Non-Hispanic White | 81869 (88.7%) | 84657 (91.7%) | 84634 (91.7%) | 85149 (92.2%) | 85221 (92.3%) |
| Pacific Islander | 200 (0.2%) | 104 (0.1%) | 104 (0.1%) | 80 (0.1%) | 64 (0.1%) |
| Unknown | 1536 (1.7%) | 1189 (1.3%) | 1055 (1.1%) | 1060 (1.1%) | 1195 (1.3%) |
| **Smoking status** |  |  |  |  |  |
| Never smoker | 33247 (36.0%) | 33732 (36.5%) | 33820 (36.6%) | 33245 (36.0%) | 30420 (32.9%) |
| Former smoker | 43359 (47.0%) | 45451 (49.2%) | 45613 (49.4%) | 45608 (49.4%) | 46066 (49.9%) |
| Current smoker | 11870 (12.9%) | 9767 (10.6%) | 9554 (10.3%) | 10207 (11.1%) | 12313 (13.3%) |
| Unknown | 3861 (4.2%) | 3386 (3.7%) | 3349 (3.6%) | 3276 (3.5%) | 3538 (3.8%) |
| **Education** |  |  |  |  |  |
| less than 12 years | 5209 (5.6%) | 4897 (5.3%) | 5177 (5.6%) | 5345 (5.8%) | 5947 (6.4%) |
| 12 years, completed high school, GED | 15892 (17.2%) | 16980 (18.4%) | 17940 (19.4%) | 19372 (21.0%) | 19939 (21.6%) |
| post-high school training | 8399 (9.1%) | 8510 (9.2%) | 9112 (9.9%) | 9569 (10.4%) | 9716 (10.5%) |
| some college | 21184 (22.9%) | 20953 (22.7%) | 21162 (22.9%) | 21320 (23.1%) | 22288 (24.1%) |
| college and postgraduate | 38610 (41.8%) | 38487 (41.7%) | 36425 (39.4%) | 34301 (37.1%) | 31702 (34.3%) |
| Unknown | 3043 (3.3%) | 2509 (2.7%) | 2520 (2.7%) | 2429 (2.6%) | 2745 (3.0%) |
| **Physical activity** |  |  |  |  |  |
| Never/Rarely | 15062 (16.3%) | 14340 (15.5%) | 15014 (16.3%) | 16012 (17.3%) | 19164 (20.8%) |
| Low (1-3 times a month) | 11382 (12.3%) | 12015 (13.0%) | 12396 (13.4%) | 13078 (14.2%) | 13959 (15.1%) |
| Moderate (1-2 times per week) | 18784 (20.3%) | 20065 (21.7%) | 20635 (22.3%) | 20881 (22.6%) | 19782 (21.4%) |
| High (≥3 times a week) | 45863 (49.7%) | 44994 (48.7%) | 43444 (47.1%) | 41456 (44.9%) | 38447 (41.6%) |
| Unknown | 1246 (1.3%) | 922 (1.0%) | 847 (0.9%) | 909 (1.0%) | 985 (1.1%) |
| **Alcohol intake category** |  |  |  |  |  |
| 0 drinks per day | 19819 (21.5%) | 19019 (20.6%) | 20243 (21.9%) | 22589 (24.5%) | 28362 (30.7%) |
| <1 drink per day | 44867 (48.6%) | 49473 (53.6%) | 51281 (55.5%) | 51496 (55.8%) | 47955 (51.9%) |
| 1-2 drinks per day | 17457 (18.9%) | 16498 (17.9%) | 14531 (15.7%) | 12623 (13.7%) | 10011 (10.8%) |
| 3-4 drinks per day | 4795 (5.2%) | 3797 (4.1%) | 3202 (3.5%) | 2984 (3.2%) | 3087 (3.3%) |
| ≥5 drinks per day | 5399 (5.8%) | 3549 (3.8%) | 3079 (3.3%) | 2644 (2.9%) | 2922 (3.2%) |
| **Body mass index category** |  |  |  |  |  |
| <18.5 kg/m^2^ | 1272 (1.4%) | 961 (1.0%) | 865 (0.9%) | 771 (0.8%) | 735 (0.8%) |
| 18.5 to <25 kg/m^2^ | 38386 (41.6%) | 35442 (38.4%) | 31508 (34.1%) | 27596 (29.9%) | 22976 (24.9%) |
| 25 to <30 kg/m^2^ | 36059 (39.1%) | 37886 (41.0%) | 39147 (42.4%) | 40184 (43.5%) | 39218 (42.5%) |
| ≥ 30 kg/m^2^ | 14045 (15.2%) | 15948 (17.3%) | 18784 (20.3%) | 21773 (23.6%) | 27328 (29.6%) |
| Unknown | 2575 (2.8%) | 2099 (2.3%) | 2032 (2.2%) | 2012 (2.2%) | 2080 (2.3%) |
| **Family history of cancer** |  |  |  |  |  |
| No | 42935 (46.5%) | 42398 (45.9%) | 42592 (46.1%) | 42723 (46.3%) | 43137 (46.7%) |
| Yes | 44444 (48.1%) | 45407 (49.2%) | 45069 (48.8%) | 44931 (48.7%) | 44322 (48.0%) |
| Unknown | 4958 (5.4%) | 4531 (4.9%) | 4675 (5.1%) | 4682 (5.1%) | 4878 (5.3%) |
| **Self-reported health status** |  |  |  |  |  |
| Excellent | 19804 (21.4%) | 17524 (19.0%) | 15975 (17.3%) | 14617 (15.8%) | 13112 (14.2%) |
| Very good | 33852 (36.7%) | 34650 (37.5%) | 33758 (36.6%) | 33078 (35.8%) | 30318 (32.8%) |
| Good | 28927 (31.3%) | 30284 (32.8%) | 32167 (34.8%) | 33576 (36.4%) | 34864 (37.8%) |
| Fair | 8271 (9.0%) | 8522 (9.2%) | 9084 (9.8%) | 9776 (10.6%) | 12619 (13.7%) |
| Unknown | 1483 (1.6%) | 1356 (1.5%) | 1352 (1.5%) | 1289 (1.4%) | 1424 (1.5%) |
| **Median total HEI-2015 score**, IQR | 70.9 (64.1-76.7) | 69.9 (63.0-75.7) | 68.9 (62.1-74.7) | 67.6 (60.6-73.6) | 65.6 (58.3-72.0) |
| **Median total energy intake (kcal)**, IQR | 1550 (1165-2025) | 1620 (1241-2106) | 1670 (1268-2172) | 1620 (1246-2126) | 1520 (1126-2020) |
| **Median dietary calcium mg/1000 kcal/day**, IQR | 417 (327-555) | 419 (335-542) | 416 (332-535) | 403 (321-519) | 405 (315-528) |
| **Median dietary fiber g/1000 kcal/day**, IQR | 11.8 (9.3-14.9) | 11.4 (9.2-14.0) | 10.8 (8.8-13.4) | 10.4 (8.4-12.9) | 10.0 (7.8-12.6) |

Abbreviations: BMI, body mass index; HEI-2015, Healthy Eating Index-2015; IQR, interquartile range; UPF, ultra-processed food

^a^Quintiles of grams classified as Nova group 4 nutrient adjusted

^b^Column percentages might not add up to exactly 100% due to rounding
