# Supplementary material for: Associations of food processing and dietary risk factors with colorectal cancer incidence in the NIH-AARP Diet and Health Study: A cohort study
Source: PLoS Med. 2026 Oct 15;23(10):e1005251. doi: 10.1371/journal.pmed.1005251 (PMC13671506; doi:10.1371/journal.pmed.1005251)
Supplement: Supplementary file 2 [file pmed.1005251.s002.docx]

**S2 Table**. Baseline characteristics of study participants who were in the NIH-AARP Diet and Health Study by inclusion in the analytic sample

| **Characteristic, N (%)** | **Included (n=461,682)** | **Excluded (n=83,492)** |
| --- | --- | --- |
| **Range UPF**, g/1000 kcal/day, male | 2.7 – 10042.6 | 11.6 – 9550.1 |
| **Range UPF**, g/1000 kcal/day, female | 9.5 – 12740.3 | 11.1 – 15448.5 |
| **Median UPF**, g/1000 kcal/day (IQR), male | 298 (201.9 - 465.1) | 291 (198.6 - 458.4) |
| **Median UPF**, g/1000 kcal/day (IQR), female | 285 (191.5 - 467.3) | 285 (190.8 - 471.5) |
| **Median age**, years (IQR) | 62.5 (57.7 - 66.6) | 64.3 (59.5 - 67.6) |
| **Sex**, n (%) |  |  |
| Male | 274269 (59.4%) | 47829 (57.3%) |
| Female | 187413 (40.6%) | 35663 (42.7%) |
| **Race/ethnicity**, n (%) |  |  |
| American Indian/Alaskan Native | 1258 (0.3%) | 278 (0.3%) |
| Asian | 5856 (1.3%) | 692 (0.8%) |
| Hispanic | 8838 (1.9%) | 1176 (1.4%) |
| Non-Hispanic Black | 17613 (3.8%) | 3165 (3.8%) |
| Non-Hispanic White | 421530 (91.3%) | 76917 (92.1%) |
| Pacific Islander | 552 (0.1%) | 82 (0.1%) |
| Unknown | 6035 (1.3%) | 1182 (1.4%) |
| **Never smoker**, n (%) | 164464 (35.6%) | 25353 (30.4%) |
| **College graduate**, n (%) | 179525 (38.9%) | 30612 (36.7%) |
| **Exercises ≥3 times a week**, n (%) | 214204 (46.4%) | 35237 (42.2%) |
| **Alcohol non-drinkers**, n (%) | 110032 (23.8%) | 23445 (28.1%) |
| **BMI ≥30 kg/m^2^**, n (%) | 97878 (21.2%) | 19269 (23.1%) |
| **Excellent self-rated health**, n (%) | 81032 (17.6%) | 8594 (10.3%) |
| **Median total energy intake,** kcal/day (IQR) | 1600 (1208.2 - 2091.1) | 1590 (1203.0 - 2098.8) |
| **Median total HEI-2015 score** (IQR) | 68.6 (61.5 - 74.7) | 68.3 (61.2 - 74.5) |
| **Median dietary fiber,** g/1000 kcal/day (IQR) | 10.9 (8.7 - 13.6) | 10.8 (8.6 - 13.4) |

Abbreviations: UPF, Ultra-processed food; HEI-2015, Healthy Eating Index-2015; IQR, interquartile range; BMI, body mass index
