# Supplementary material for: Associations of food processing and dietary risk factors with colorectal cancer incidence in the NIH-AARP Diet and Health Study: A cohort study
Source: PLoS Med. 2026 Oct 15;23(10):e1005251. doi: 10.1371/journal.pmed.1005251 (PMC13671506; doi:10.1371/journal.pmed.1005251)
Supplement: Supplementary file 3 [file pmed.1005251.s003.docx]

**S3 Table**. Association of ultra-processed food intake (quintiles of nutrient-adjusted g/1000 kcal/day) with colorectal cancer risk by anatomic location in male participants in the NIH-AARP Diet and Health Study (N=274,269)

|  | UPF Q1  (N=54854) | UPF Q2  (N=54854) | UPF Q3  (N=54853) | UPF Q4  (N=54854) | UPF Q5  (N=54854) | P-trend^b^ |
| --- | --- | --- | --- | --- | --- | --- |
| Colorectal cancer^a^ |  |  |  |  |  |  |
| No. of cases | 1380 | 1216 | 1244 | 1267 | 1307 |  |
| Base model, HR (95% CI)^c^ | 1.00 (ref) | 0.87 (0.81 to 0.94) | 0.90 (0.84 to 0.97) | 0.93 (0.86 to 1.00) | 1.00 (0.92 to 1.07) | .11 |
| MV model, HR (95% CI)^d^ | 1.00 (ref) | 0.88 (0.82 to 0.95) | 0.91 (0.84 to 0.98) | 0.93 (0.86 to 1.00) | 0.97 (0.90 to 1.04) | .56 |
| + HEI and dietary factors, HR (95% CI) ^e^ | 1.00 (ref) | 0.88 (0.81 to 0.95) | 0.90 (0.83 to 0.97) | 0.90 (0.84 to 0.98) | 0.94 (0.86 to 1.01) | .77 |
| + BMI, HR (95% CI) ^f^ | 1.00 (ref) | 0.88 (0.82 to 0.95) | 0.90 (0.84 to 0.98) | 0.92 (0.85 to 0.99) | 0.95 (0.88 to 1.02) | .99 |
| Distal colon |  |  |  |  |  |  |
| No. of cases | 370 | 338 | 375 | 337 | 354 |  |
| Base model, HR (95% CI)^c^ | 1.00 (ref) | 0.90 (0.78 to 1.04) | 1.00 (0.87 to 1.16) | 0.90 (0.78 to 1.04) | 0.97 (0.84 to 1.13) | .98 |
| MV model, HR (95% CI)^d^ | 1.00 (ref) | 0.92 (0.79 to 1.06) | 1.01 (0.87 to 1.17) | 0.90 (0.77 to 1.04) | 0.93 (0.80 to 1.08) | .43 |
| + HEI and dietary factors, HR (95% CI) ^e^ | 1.00 (ref) | 0.90 (0.78 to 1.05) | 0.98 (0.85 to 1.14) | 0.86 (0.74 to 1.00) | 0.88 (0.76 to 1.03) | .13 |
| + BMI, HR (95% CI) ^f^ | 1.00 (ref) | 0.91 (0.79 to 1.06) | 0.99 (0.86 to 1.15) | 0.88 (0.75 to 1.02) | 0.90 (0.77 to 1.04) | .17 |
| Proximal colon |  |  |  |  |  |  |
| No. of cases | 585 | 557 | 516 | 567 | 582 |  |
| Base model, HR (95% CI)^c^ | 1.00 (ref) | 0.95 (0.85 to 1.07) | 0.90 (0.80 to 1.01) | 1.00 (0.89 to 1.12) | 1.09 (0.97 to 1.22) | .019 |
| MV model, HR (95% CI)^d^ | 1.00 (ref) | 0.95 (0.85 to 1.07) | 0.90 (0.79 to 1.01) | 0.99 (0.88 to 1.11) | 1.05 (0.93 to 1.18) | .10 |
| + HEI and dietary factors, HR (95% CI) ^e^ | 1.00 (ref) | 0.95 (0.84 to 1.07) | 0.89 (0.79 to 1.00) | 0.98 (0.87 to 1.10) | 1.03 (0.92 to 1.16) | .18 |
| + BMI, HR (95% CI) ^f^ | 1.00 (ref) | 0.95 (0.84 to 1.07) | 0.89 (0.79 to 1.00) | 0.98 (0.87 to 1.10) | 1.02 (0.91 to 1.15) | .26 |
| Rectal |  |  |  |  |  |  |
| No. of cases | 375 | 291 | 329 | 330 | 335 |  |
| Base model, HR (95% CI)^c^ | 1.00 (ref) | 0.76 (0.66 to 0.89) | 0.87 (0.75 to 1.00) | 0.87 (0.75 to 1.01) | 0.91 (0.78 to 1.05) | .91 |
| MV model, HR (95% CI)^d^ | 1.00 (ref) | 0.79 (0.67 to 0.92) | 0.89 (0.77 to 1.03) | 0.89 (0.77 to 1.03) | 0.91 (0.78 to 1.05) | .96 |
| + HEI and dietary factors, HR (95% CI) ^e^ | 1.00 (ref) | 0.78 (0.66 to 0.90) | 0.87 (0.75 to 1.01) | 0.86 (0.74 to 1.00) | 0.86 (0.74 to 1.00) | .45 |
| + BMI, HR (95% CI) ^f^ | 1.00 (ref) | 0.79 (0.67 to 0.92) | 0.89 (0.77 to 1.04) | 0.89 (0.77 to 1.04) | 0.91 (0.78 to 1.06) | .98 |

^a^Cases with cancer sites of C188, C189, and C260 are included in the overall analysis of 10075 cases but are censored at diagnosis date in the anatomic location analysis

^b^P-trend is based on the Wald test, by assigning each quintile to its median value and treating as a continuous variable in the Cox proportional hazards regression model

^c^Adjusted for age in years (underlying time metric), sex (male/female), and total daily energy (kcal/day) for nutrient density adjustment

^d^ Adjusted for age in years (underlying time metric), total daily energy (kcal/day), race/ethnicity (American Indian/Alaskan Native, Asian, Hispanic, Non-Hispanic Black, Non-Hispanic White, Pacific Islander, unknown), smoking status (never, former, current, unknown) by intensity (cigarettes per day: 1-10, 11-20, 21-30, 31-40, 41-60, 61+) and time since cessation (10+, 5-9, 1-4 years ago, within the last year), education level (11 years or less; 12 years, completed high school, or GED; post-high school training; some college; college and post graduate; unknown), physical activity level (never/rarely, low, moderate, high, unknown), alcohol intake (0, < 1, 1-2, 3-4, 5+ standard drinks/day), family history of cancer (yes, no, unknown), and self-reported health status (excellent, very good, good, fair, unknown)

^e^ MV model further adjusted for Healthy Eating Index (HEI)-2015 score quartiles (quartile 1: 21.5 to <60.6, quartile 2: 60.6 to <67.7, quartile 3: 67.7 to <74.0, quartile 4: 74.0 to 97.5), nutrient-density adjusted dietary calcium quintiles (quintile 1: 79.2 to <303.6, quintile 2: 303.6 to <367.0, quintile 3: 367.0 to <438.5, quintile 4: 438.5 to <551.3, quintile 5: 551.3 to 2312.8 mg/1000 kcal/day), and nutrient-density adjusted dietary fiber quintiles (quintile 1: 0.56 to <7.93, quintile 2: 7.93 to <9.69, quintile 3: 9.69 to <11.44, quintile 4: 11.44 to <13.89, quintile 5: 13.89 to 51.98 g/1000 kcal/day)

^f^ MV model further adjusted for body mass index category (<18.5 kg/m2, 18.5 to <25 kg/m2, 25 to <30 kg/m2, ≥30 kg/m2, unknown)

Abbreviations: BMI, body mass index; HEI-2015, Healthy Eating Index-2015; IQR, interquartile range; MV, multivariable; Q, quintile; UPF, ultra-processed food
