# Supplementary material for: Associations of food processing and dietary risk factors with colorectal cancer incidence in the NIH-AARP Diet and Health Study: A cohort study
Source: PLoS Med. 2026 Oct 15;23(10):e1005251. doi: 10.1371/journal.pmed.1005251 (PMC13671506; doi:10.1371/journal.pmed.1005251)
Supplement: Supplementary file 4 [file pmed.1005251.s004.docx]

**S4 Table**. Association of ultra-processed food intake (quintiles of nutrient-adjusted g/1000 kcal/day) with colorectal cancer risk by anatomic location in female participants in the NIH-AARP Diet and Health Study (N=187,413)

|  | UPF Q1  (N=37483) | UPF Q1  (N=37482) | UPF Q1  (N=37483) | UPF Q1  (N=37482) | UPF Q1  (N=37483) | P-trend^b^ |
| --- | --- | --- | --- | --- | --- | --- |
| Colorectal cancer^a^ |  |  |  |  |  |  |
| No. of cases | 772 | 730 | 673 | 764 | 722 |  |
| Base model, HR (95% CI)^c^ | 1.00 (ref) | 0.94 (0.85 to 1.04) | 0.87 (0.79 to 0.97) | 1.02 (0.92 to 1.13) | 1.03 (0.93 to 1.14) | .10 |
| MV model, HR (95% CI)^d^ | 1.00 (ref) | 0.95 (0.86 to 1.06) | 0.88 (0.79 to 0.98) | 1.01 (0.91 to 1.11) | 0.97 (0.88 to 1.08) | .78 |
| + HEI and dietary factors, HR (95% CI) ^e^ | 1.00 (ref) | 0.96 (0.86 to 1.06) | 0.88 (0.79 to 0.98) | 1.00 (0.90 to 1.11) | 0.95 (0.86 to 1.06) | .88 |
| + BMI, HR (95% CI) ^f^ | 1.00 (ref) | 0.95 (0.86 to 1.05) | 0.87 (0.79 to 0.97) | 0.99 (0.90 to 1.10) | 0.95 (0.86 to 1.06) | .92 |
| Distal colon |  |  |  |  |  |  |
| No. of cases | 185 | 162 | 150 | 185 | 177 |  |
| Base model, HR (95% CI)^c^ | 1.00 (ref) | 0.87 (0.70 to 1.07) | 0.80 (0.64 to 0.99) | 1.00 (0.82 to 1.23) | 1.00 (0.81 to 1.23) | .32 |
| MV model, HR (95% CI)^d^ | 1.00 (ref) | 0.89 (0.72 to 1.10) | 0.82 (0.66 to 1.02) | 1.01 (0.82 to 1.24) | 0.96 (0.78 to 1.18) | .72 |
| + HEI and dietary factors, HR (95% CI) ^e^ | 1.00 (ref) | 0.90 (0.72 to 1.11) | 0.82 (0.66 to 1.02) | 0.99 (0.80 to 1.22) | 0.94 (0.76 to 1.16) | .89 |
| + BMI, HR (95% CI) ^f^ | 1.00 (ref) | 0.89 (0.72 to 1.09) | 0.81 (0.65 to 1.01) | 0.98 (0.80 to 1.21) | 0.92 (0.75 to 1.14) | .97 |
| Proximal colon |  |  |  |  |  |  |
| No. of cases | 409 | 415 | 365 | 412 | 379 |  |
| Base model, HR (95% CI)^c^ | 1.00 (ref) | 1.01 (0.88 to 1.16) | 0.90 (0.78 to 1.04) | 1.05 (0.92 to 1.21) | 1.05 (0.92 to 1.21) | .26 |
| MV model, HR (95% CI)^d^ | 1.00 (ref) | 1.02 (0.89 to 1.16) | 0.90 (0.78 to 1.03) | 1.03 (0.90 to 1.19) | 0.99 (0.86 to 1.14) | .93 |
| + HEI and dietary factors, HR (95% CI) ^e^ | 1.00 (ref) | 1.02 (0.89 to 1.17) | 0.90 (0.78 to 1.04) | 1.02 (0.89 to 1.18) | 0.97 (0.84 to 1.12) | .76 |
| + BMI, HR (95% CI) ^f^ | 1.00 (ref) | 1.01 (0.88 to 1.16) | 0.89 (0.78 to 1.03) | 1.03 (0.89 to 1.18) | 0.98 (0.85 to 1.13) | .98 |
| Rectal |  |  |  |  |  |  |
| No. of cases | 160 | 133 | 144 | 148 | 144 |  |
| Base model, HR (95% CI)^c^ | 1.00 (ref) | 0.83 (0.66 to 1.04) | 0.89 (0.71 to 1.12) | 0.93 (0.74 to 1.16) | 0.94 (0.75 to 1.18) | .85 |
| MV model, HR (95% CI)^d^ | 1.00 (ref) | 0.85 (0.67 to 1.07) | 0.92 (0.73 to 1.15) | 0.94 (0.75 to 1.18) | 0.92 (0.73 to 1.16) | .90 |
| + HEI and dietary factors, HR (95% CI) ^e^ | 1.00 (ref) | 0.85 (0.68 to 1.08) | 0.93 (0.74 to 1.17) | 0.95 (0.75 to 1.19) | 0.93 (0.73 to 1.17) | .91 |
| + BMI, HR (95% CI) ^f^ | 1.00 (ref) | 0.85 (0.67 to 1.07) | 0.91 (0.73 to 1.15) | 0.92 (0.74 to 1.16) | 0.90 (0.71 to 1.13) | .71 |

^a^Cases with cancer sites of C188, C189, and C260 are included in the overall analysis of 3661 cases but are censored at diagnosis date in the anatomic location analysis

^b^ P-trend is based on the Wald test, by assigning each quintile to its median value and treating as a continuous variable in the Cox proportional hazards regression model

^c^Adjusted for age in years (underlying time metric) and total daily energy (kcal/day) for nutrient density adjustment

^d^ Adjusted for age in years (underlying time metric), total daily energy (kcal/day), race/ethnicity (American Indian/Alaskan Native, Asian, Hispanic, Non-Hispanic Black, Non-Hispanic White, Pacific Islander, unknown), smoking status (never, former, current, unknown) by intensity (cigarettes per day: 1-10, 11-20, 21-30, 31-40, 41-60, 61+) and time since cessation (10+, 5-9, 1-4 years ago, within the last year), education level (11 years or less; 12 years, completed high school, or GED; post-high school training; some college; college and post graduate; unknown), physical activity level (never/rarely, low, moderate, high, unknown), alcohol intake (0, < 1, 1-2, 3-4, 5+ standard drinks/day), family history of cancer (yes, no, unknown), and self-reported health status (excellent, very good, good, fair, unknown)

^e^ MV model further adjusted for Healthy Eating Index (HEI)-2015 score quartiles (quartile 1: 25.9 to <63.0, quartile 2: 63.0 to <69.8, quartile 3: 69.8 to <75.6, quartile 4: 75.6 to 95.8), nutrient-density adjusted dietary calcium quintiles (quintile 1: 53.9 to <318.1, quintile 2: 318.1 to <392.6, quintile 3: 392.6 to <476.6, quintile 4: 476.6 to <605.2, quintile 5: 605.2 to 2321.7 mg/1000 kcal/day), and nutrient-density adjusted dietary fiber quintiles (quintile 1: 1.01 to <8.57, quintile 2: 8.57 to <10.47, quintile 3: 10.47 to <12.34, quintile 4: 12.34 to <14.93, quintile 5: 14.93 to 57.17 g/1000 kcal/day).

^f^ MV model further adjusted for body mass index category (<18.5 kg/m2, 18.5 to <25 kg/m2, 25 to <30 kg/m2, ≥30 kg/m2, unknown)

Abbreviations: BMI, body mass index; GED, General Equivalency Diploma; HEI-2015, Healthy Eating Index-2015; IQR, interquartile range; MV, multivariable; Q, quintile; UPF, ultra-processed food
