# Supplementary material for: Associations of food processing and dietary risk factors with colorectal cancer incidence in the NIH-AARP Diet and Health Study: A cohort study
Source: PLoS Med. 2026 Oct 15;23(10):e1005251. doi: 10.1371/journal.pmed.1005251 (PMC13671506; doi:10.1371/journal.pmed.1005251)
Supplement: Supplementary file 5 [file pmed.1005251.s005.docx]

**S5 Table**. Association of ultra-processed food intake (quintiles of nutrient-adjusted g/1000 kcal/day) with colorectal cancer risk stratified by BMI category

|  | HR (95% CI) | | | | |
| --- | --- | --- | --- | --- | --- |
| BMI category | UPF Q1 | UPF Q2 | UPF Q3 | UPF Q4 | UPF Q5 |
| < 18.5 kg/m^2^ | 1.00 | 0.78 (0.42 to 1.44) | 1.22 (0.69 to 2.13) | 0.69 (0.35 to 1.39) | 0.91 (0.47 to 1.77) |
| 18.5 to <25 kg/m^2^ | 1.00 | 0.89 (0.81 to 0.99) | 0.85 (0.76 to 0.94) | 0.90 (0.81 to 1.01) | 0.91 (0.81 to 1.02) |
| 25 to <30 kg/m^2^ | 1.00 | 0.93 (0.85 to 1.02) | 0.90 (0.81 to 0.99) | 0.97 (0.89 to 1.07) | 0.92 (0.83 to 1.01) |
| ≥ 30 kg/m^2^ | 1.00 | 0.87 (0.76 to 1.01) | 0.91 (0.79 to 1.04) | 0.89 (0.78 to 1.02) | 0.98 (0.86 to 1.11) |

^a^ Adjusted for age in years (underlying time metric), total daily energy (kcal/day), sex (male, female), race/ethnicity (American Indian/Alaskan Native, Asian, Hispanic, Non-Hispanic Black, Non-Hispanic White, Pacific Islander, unknown), smoking status (never, former, current, unknown) by intensity (cigarettes per day: 1-10, 11-20, 21-30, 31-40, 41-60, 61+) and time since cessation (10+, 5-9, 1-4 years ago, within the last year), education level (11 years or less; 12 years, completed high school, or GED; post-high school training; some college; college and post graduate; unknown), physical activity level (never/rarely, low, moderate, high, unknown), alcohol intake (0, < 1, 1-2, 3-4, 5+ standard drinks/day), family history of cancer (yes, no, unknown), and self-reported health status (excellent, very good, good, fair, unknown)

Abbreviations: BMI, body mass index; GED, General Equivalency Diploma; HEI-2015, Healthy Eating Index-2015; IQR, interquartile range; MV, multivariable; Q, quintile; UPF, ultra-processed food
