# Supplementary material for: Associations of food processing and dietary risk factors with colorectal cancer incidence in the NIH-AARP Diet and Health Study: A cohort study
Source: PLoS Med. 2026 Oct 15;23(10):e1005251. doi: 10.1371/journal.pmed.1005251 (PMC13671506; doi:10.1371/journal.pmed.1005251)
Supplement: Supplementary file 6 [file pmed.1005251.s006.docx]

**S6 Table**. Dietary calcium, dairy, whole grain, fiber, and meat intake by Nova group in the NIH-AARP Diet and Health Study (N=461,682)

| Nutrient/Food group (%), mean (SD) | Nova 1 | Nova 2 | Nova 3 | Nova 4 |
| --- | --- | --- | --- | --- |
| Dietary Calcium | 55.5 (15.5) | 1.6 (2.9) | 11.3 (8.0) | 31.7 (12.4) |
| Dairy | 60.1 (25.7) | 0.9 (4.9) | 15.5 (14.5) | 23.5 (17.3) |
| Whole Grain | 17.1 (15.8) | 0 (0) | 0 (0) | 82.8 (16.0) |
| Fiber | 58.3 (12.7) | 0.2 (0.2) | 8.0 (4.6) | 33.5 (12.9) |
| Red meat | 98.4 (4.6) | 0 (0) | 0.02 (0.1) | 1.4 (1.7) |
| Processed meat | 0 (0) | 0 (0) | 25.5 (17.8) | 74.3 (18.1) |
| Total meat | 71.6 (14.0) | 0 (0) | 10.9 (8.21) | 17.4 (12.4) |

Nova 1=Unprocessed or minimally processed foods; Nova 2=Processed culinary ingredients; Nova 3=Processed foods; Nova 4=Ultra-processed foods.

Abbreviation: SD, standard deviation
