# Supplementary material for: Associations of food processing and dietary risk factors with colorectal cancer incidence in the NIH-AARP Diet and Health Study: A cohort study
Source: PLoS Med. 2026 Oct 15;23(10):e1005251. doi: 10.1371/journal.pmed.1005251 (PMC13671506; doi:10.1371/journal.pmed.1005251)
Supplement: Supplementary file 8 [file pmed.1005251.s008.docx]

**S8 Table**. Association of UPF intake (sex-specific quintiles of % grams/day) with colorectal cancer risk in the NIH-AARP Diet and Health Study (N=461,682)

| Cancer type | N^a^ | UPF Q1  (N=92337) | UPF Q2  (N=92336) | UPF Q3  (N=92336) | UPF Q4  (N=92336) | UPF Q5  (N=92337) | P-trend^b^ |
| --- | --- | --- | --- | --- | --- | --- | --- |
| Colorectal cancer |  |  |  |  |  |  |  |
| No. of cases | 10075 | 2084 | 1947 | 1963 | 2030 | 2051 |  |
| Base model, HR (95% CI)^c^ |  | 1.00 (ref) | 0.93 (0.87 to 0.99) | 0.94 (0.88 to 1.00) | 0.98 (0.92 to 1.05) | 1.03 (0.97 to 1.10) | .02 |
| MV model, HR (95% CI)^d^ |  | 1.00 (ref) | 0.94 (0.88 to 1.00) | 0.95 (0.89 to 1.01) | 0.98 (0.92 to 1.05) | 1.01 (0.95 to 1.07) | .21 |
| + HEI and dietary factors, HR (95% CI) ^e^ |  | 1.00 (ref) | 0.93 (0.87 to 0.99) | 0.93 (0.87 to 0.99) | 0.95 (0.89 to 1.01) | 0.94 (0.88 to 1.01) | .34 |
| + BMI, HR (95% CI) ^f^ |  | 1.00 (ref) | 0.94 (0.88 to 1.00) | 0.94 (0.89 to 1.01) | 0.97 (0.91 to 1.04) | 0.99 (0.93 to 1.05) | .56 |
| Distal colon |  |  |  |  |  |  |  |
| No. of cases | 2633 | 546 | 492 | 514 | 549 | 532 |  |
| Base model, HR (95% CI)^c^ |  | 1.00 (ref) | 0.89 (0.78 to 1.00) | 0.92 (0.82 to 1.04) | 0.99 (0.88 to 1.12) | 0.98 (0.87 to 1.11) | .51 |
| MV model, HR (95% CI)^d^ |  | 1.00 (ref) | 0.90 (0.80 to 1.02) | 0.93 (0.83 to 1.06) | 0.98 (0.87 to 1.11) | 0.94 (0.83 to 1.06) | .80 |
| + HEI and dietary factors, HR (95% CI) ^e^ |  | 1.00 (ref) | 0.89 (0.78 to 1.00) | 0.90 (0.80 to 1.02) | 0.93 (0.82 to 1.05) | 0.85 (0.75 to 0.97) | .07 |
| + BMI, HR (95% CI) ^f^ |  | 1.00 (ref) | 0.90 (0.79 to 1.01) | 0.92 (0.82 to 1.04) | 0.96 (0.85 to 1.09) | 0.91 (0.80 to 1.03) | .42 |
| Proximal colon |  |  |  |  |  |  |  |
| No. of cases | 4787 | 982 | 927 | 923 | 958 | 997 |  |
| Base model, HR (95% CI)^c^ |  | 1.00 (ref) | 0.94 (0.86 to 1.03) | 0.96 (0.87 to 1.05) | 1.01 (0.93 to 1.11) | 1.12 (1.02 to 1.22) | <.001 |
| MV model, HR (95% CI)^d^ |  | 1.00 (ref) | 0.95 (0.86 to 1.03) | 0.95 (0.87 to 1.04) | 1.00 (0.91 to 1.09) | 1.08 (0.98 to 1.18) | .02 |
| + HEI and dietary factors, HR (95% CI) ^e^ |  | 1.00 (ref) | 0.94 (0.86 to 1.03) | 0.94 (0.86 to 1.03) | 0.97 (0.89 to 1.07) | 1.03 (0.93 to 1.13) | .22 |
| + BMI, HR (95% CI) ^f^ |  | 1.00 (ref) | 0.94 (0.86 to 1.03) | 0.95 (0.86 to 1.04) | 0.99 (0.90 to 1.08) | 1.06 (0.96 to 1.16) | .05 |
| Rectal |  |  |  |  |  |  |  |
| No. of cases | 2389 | 492 | 472 | 484 | 474 | 467 |  |
| Base model, HR (95% CI)^c^ |  | 1.00 (ref) | 0.95 (0.83 to 1.07) | 0.97 (0.85 to 1.10) | 0.95 (0.84 to 1.08) | 0.96 (0.84 to 1.09) | .65 |
| MV model, HR (95% CI)^d^ |  | 1.00 (ref) | 0.97 (0.86 to 1.11) | 1.00 (0.88 to 1.14) | 0.99 (0.87 to 1.12) | 0.98 (0.86 to 1.11) | .77 |
| + HEI and dietary factors, HR (95% CI) ^e^ |  | 1.00 (ref) | 0.96 (0.85 to 1.10) | 0.98 (0.86 to 1.11) | 0.94 (0.83 to 1.07) | 0.90 (0.79 to 1.03) | .11 |
| + BMI, HR (95% CI) ^f^ |  | 1.00 (ref) | 0.98 (0.86 to 1.11) | 1.00 (0.88 to 1.14) | 0.98 (0.86 to 1.12) | 0.97 (0.85 to 1.11) | .70 |

Abbreviations: BMI, body mass index, calculated as weight in kilograms divided by height in meters squared; CI, confidence interval; HEI, Healthy Eating Index 2015; HR, hazard ratio; MV, multivariable

^a^Cases with cancer sites of C188, C189, and C260 are included in the overall analysis of 10075 cases but are censored at diagnosis date in the anatomic location analysis

^b^P-trend is based on the Wald test, by assigning each quintile to its median value and treating as a continuous variable in the Cox proportional hazards regression model

^c^Adjusted for age in years (underlying time metric), sex (male/female), and total daily energy (kcal/day) for nutrient density adjustment

^d^Adjusted for age in years (underlying time metric), total daily energy (kcal/day), sex (male, female), race/ethnicity (American Indian/Alaskan Native, Asian, Hispanic, Non-Hispanic Black, Non-Hispanic White, Pacific Islander, unknown), smoking status (never, former, current, unknown) by intensity (cigarettes per day: 1-10, 11-20, 21-30, 31-40, 41-60, 61+) and time since cessation (10+, 5-9, 1-4 years ago, within the last year), education level (11 years or less; 12 years, completed high school, or GED; post-high school training; some college; college and post graduate; unknown), physical activity level (never/rarely, low, moderate, high, unknown), alcohol intake (0, < 1, 1-2, 3-4, 5+ standard drinks/day), family history of cancer (yes, no, unknown), and self-reported health status (excellent, very good, good, fair, unknown)

^e^MV model further adjusted for Healthy Eating Index (HEI)-2015 score quartiles (males - quartile 1: 21.5 to <60.6, quartile 2: 60.6 to <67.7, quartile 3: 67.7 to <74.0, quartile 4: 74.0 to 97.5; females - quartile 1: 25.9 to <63.0, quartile 2: 63.0 to <69.8, quartile 3: 69.8 to <75.6, quartile 4: 75.6 to 95.8), dietary calcium quintiles (males - quintile 1: 79.2 to <303.6, quintile 2: 303.6 to <367.0, quintile 3: 367.0 to <438.5, quintile 4: 438.5 to <551.3, quintile 5: 551.3 to 2312.8 mg/1000 kcal/day; females - quintile 1: 53.9 to <318.1, quintile 2: 318.1 to <392.6, quintile 3: 392.6 to <476.6, quintile 4: 476.6 to <605.2, quintile 5: 605.2 to 2321.7 mg/1000 kcal/day), and dietary fiber quintiles (males - quintile 1: 0.56 to <7.93, quintile 2: 7.93 to <9.69, quintile 3: 9.69 to <11.44, quintile 4: 11.44 to <13.89, quintile 5: 13.89 to 51.98 g/1000 kcal/day; females - quintile 1: 1.01 to <8.57, quintile 2: 8.57 to <10.47, quintile 3: 10.47 to <12.34, quintile 4: 12.34 to <14.93, quintile 5: 14.93 to 57.17 g/1000 kcal/day)

^f^MV model further adjusted for body mass index category (<18.5 kg/m2, 18.5 to <25 kg/m2, 25 to <30 kg/m2, ≥30 kg/m2, unknown)

Abbreviations: BMI, body mass index; GED, General Equivalency Diploma; HEI-2015, Healthy Eating Index-2015; IQR, interquartile range; MV, multivariable; Q, quintile; UPF, ultra-processed food
