# Supplementary material for: Associations of food processing and dietary risk factors with colorectal cancer incidence in the NIH-AARP Diet and Health Study: A cohort study
Source: PLoS Med. 2026 Oct 15;23(10):e1005251. doi: 10.1371/journal.pmed.1005251 (PMC13671506; doi:10.1371/journal.pmed.1005251)
Supplement: Supplementary file 9 [file pmed.1005251.s009.docx]

**S9 Table**. Absolute intakes and relative contributions of subgroups to UPF intake (Nova group 4) in NIH-AARP (N=461,682)

| Subgroup | Mean (SD) g/day | Mean (SD) % within Nova group 4 (% g/day) | Mean (SD) % within total diet (% g/day) | Mean (SD) kcal/day | Mean (SD) % within Nova group 4 (% kcal/day) | Mean (SD) % within total diet (% kcal/day) |
| --- | --- | --- | --- | --- | --- | --- |
| Reconstituted meat or fish products | 20.1 (21.9) | 4.0 (4.3) | 0.8 (0.8) | 48.9 (50.8) | 6.5 (5.3) | 2.8 (2.4) |
| Bread | 57.3 (43.2) | 12.3 (10.1) | 2.2 (1.8) | 161.4 (120.0) | 22.0 (12.0) | 9.4 (5.8) |
| Baked goods and candy^a^ | 34.5 (35.4) | 7.0 (6.8) | 1.3 (1.3) | 118.8 (134.1) | 14.8 (10.8) | 6.6 (5.7) |
| Frozen desserts and milk drinks^b^ | 46.9 (58.1) | 9.4 (11.2) | 1.8 (2.2) | 60.6 (74.2) | 8.2 (8.8) | 3.5 (3.9) |
| Breakfast cereals | 15.2 (16.1) | 3.6 (4.7) | 0.6 (0.7) | 50.4 (56.1) | 7.5 (8.4) | 3.1 (3.5) |
| Salty snacks and French fries^c^ | 15.4 (17.2) | 3.0 (3.4) | 0.6 (0.7) | 63.2 (73.7) | 8.2 (7.2) | 3.5 (3.4) |
| Ready to eat meals^d^ | 6.7 (9.0) | 1.5 (2.1) | 0.3 (0.3) | 8.6 (11.7) | 1.3 (1.7) | 0.5 (0.6) |
| Instant and canned soups | 19.6 (23.7) | 4.4 (5.4) | 0.7 (0.8) | 12.3 (14.9) | 1.8 (2.2) | 0.7 (0.8) |
| Sauces, dressings, and gravies | 32.3 (27.3) | 7.1 (6.6) | 1.2 (1.0) | 79.3 (68.8) | 11.3 (8.3) | 4.7 (3.5) |
| Carbonated soft drinks | 259.3 (469.4) | 27.5 (25.9) | 8.6 (12.3) | 36.2 (109.0) | 3.8 (8.7) | 1.9 (4.9) |
| Other sweetened beverages | 140.2 (289.3) | 16.8 (21.1) | 4.7 (7.9) | 24.5 (74.1) | 2.9 (6.2) | 1.3 (3.2) |
| Other group 4 | 16.6 (12.5) | 3.6 (3.1) | 0.6 (0.5) | 84.6 (67.0) | 11.6 (7.2) | 4.9 (3.1) |

^a^ Baked goods and candy combine Nova subgroups 24 (cakes, cookies, and pies), 26 (desserts), and 29 (sweet snacks) [24]

^b^ Frozen desserts and milk drinks combine Nova subgroups 25 (ice cream and ice pops) and 37 (milk drinks).

^c^ Salty snacks and French fries combine Nova subgroups 28 (salty snacks) and 33 (French fries and other potato products).

^d^ Ready to eat meals combine Nova subgroups 30 (frozen and shelf-stable meals), 31 (pizza) and 32 (sandwiches and hamburgers on buns).

Abbreviations: SD, standard deviation; UPF, ultra-processed food
