# Supplementary material for: Associations of food processing and dietary risk factors with colorectal cancer incidence in the NIH-AARP Diet and Health Study: A cohort study
Source: PLoS Med. 2026 Oct 15;23(10):e1005251. doi: 10.1371/journal.pmed.1005251 (PMC13671506; doi:10.1371/journal.pmed.1005251)
Supplement: Supplementary file 10 [file pmed.1005251.s010.docx]

**SFig 1**. Intake of ultra-processed food subgroups and colorectal cancer risk in the NIH-AARP Diet and Health Study (N=461,682)

The forest plot shows HRs (95% CIs) for UPF subgroup Q5 (g/1000 kcal/day), with Q1 as the reference, estimated using a Cox proportional hazard regression model adjusted for age in years (underlying time metric), total daily energy (kcal/day), sex (male, female), race/ethnicity (American Indian/Alaskan Native, Asian, Hispanic, Non-Hispanic Black, Non-Hispanic White, Pacific Islander, unknown), smoking status (never, former, current, unknown) by intensity (cigarettes per day: 1-10, 11-20, 21-30, 31-40, 41-60, 61+) and time since cessation (10+, 5-9, 1-4 years ago, within the last year), education level (11 years or less; 12 years, completed high school, or GED; post-high school training; some college; college and post graduate; unknown), physical activity level (never/rarely, low, moderate, high, unknown), alcohol intake (0, < 1, 1-2, 3-4, 5+ standard drinks/day), family history of cancer (yes, no, unknown), and self-reported health status (excellent, very good, good, fair, unknown), and all other UPF subgroups. Dietary factors included nutrient-density adjusted dietary calcium (quintiles) and nutrient-density adjusted dietary fiber (quintiles). The P-trend was estimated by assigning each UPF subgroup quintile to its median value and treating it as a continuous variable.

Abbreviations: BMI, body mass index; GED, General Equivalency Diploma; HEI-2015, Healthy Eating Index-2015; IQR, interquartile range; MV, multivariable; Q, quintile; UPF, ultra-processed food
